# SNAP47 Silencing Impairs the Morphology and Neurotransmission of Hippocampal GABAergic Neurons

**DOI:** 10.1007/s12035-026-05907-8

**Published:** 2026-05-15

**Authors:** Zhengzheng He, Marcial Camacho, Thorsten Trimbuch, Heike Heilmann, Christian Rosenmund, Imre Vida, Agnieszka Münster-Wandowski

**Affiliations:** 1 Institute for Integrative Neuroanatomy, Charité - Universitätsmedizin Berlin, Corporate Member of Freie Universität Berlin, Humboldt-Universität Zu Berlin, and Berlin Institute of Health, Berlin, Germany; 2 Institute of Neurophysiology, Charité - Universitätsmedizin Berlin, Corporate Member of Freie Universität Berlin, Humboldt-Universität Zu Berlin, and Berlin Institute of Health, Berlin, Germany; 3 Department of Physical Medicine and Pharmacology, University of La Laguna, San Cristóbal de La Laguna, Spain; 4 NeuroCure Cluster of Excellence, Charité - Universitätsmedizin Berlin, Campus Mitte, Berlin, Germany

**Keywords:** SNAP47, Hippocampus, GABAergic neurons, Autaptic neuronal culture

## Abstract

Synaptosomal-associated protein 47 (SNAP47), a non-canonical SNARE (soluble N-ethylmaleimide-sensitive-factor attachment receptor) protein, is highly expressed in neuronal tissue and exhibits broad cytoplasmic distribution at the cellular level. However, SNAP47 does not directly participate in exocytosis or the recycling of synaptic vesicles (SVs), and its precise physiological function remains elusive. Our previous study revealed that SNAP47 is strongly expressed in GABAergic interneurons (INs) in the hippocampus. Therefore, we created a lentiviral vector carrying a small hairpin RNA (shRNA) to knockdown (KD) SNAP47 and applied this virus to autaptic hippocampal neuronal cultures. To identify GABAergic INs, cultures were prepared using transgenic mice expressing yellow fluorescent protein (YFP)-Venus under the control of the vesicular GABA transporter (VGAT) promoter. SNAP47 KD efficiently reduced the expression of the endogenous SNAP47 in the majority of neurons in autaptic cultures, significantly decreasing the somatic level of the protein. Morphological analysis of INs revealed that SNAP47 KD impacted the morphology with significant reduction in dendrite length. Furthermore, expression of both pre- and postsynaptic markers, was reduced in these neurons. Convergent to these morphological finding, physiological changes were also observed in whole-cell recordings, in particular a decrease in the amplitude and frequency of miniature inhibitory postsynaptic currents (mIPSCs), indicating an alteration in inhibitory synaptic transmission. In contrast, the somato-dendritic morphology and synaptic transmission of YFP-Venus-negative, putative glutamatergic excitatory neurons appeared to remain unaffected. In summary, our results demonstrate that reduced SNAP47 protein expression directly impacts the morphology of GABAergic neurons and their synaptic physiology.

**Supplementary Information:**

The online version contains supplementary material available at 10.1007/s12035-026-05907-8.

## Introduction

SNAP47 (the 47 kDa isoform of synaptosomal-associated protein) is the first SNARE protein with a lipid-binding domain identified in mammals and was first described by Holt et al. (2006). Mouse SNAP47, encodes 413 amino acid protein with a calculated molecular mass of 46.5 kDa and showed ubiquitous expression in several mouse tissues, with high levels in the brain (Holt et al. 2006). Initially, SNAP47 protein was isolated from a fraction enriched in small vesicles, but it appears to have a broad intracellular localization including SVs but also other intracellular membrane pools. Over the past years, SNAP47 has attracted substantial interest, as it is expressed much earlier than synaptic proteins during brain development and has been classified as a non-canonical SNARE protein that is not directly involved in synaptic vesicle exocytosis (Zhang et al. 2002; Pan et al. 2009; Kuster et al. 2015). Nevertheless, its function in neurons remains only partially understood.

Kuster et al. (2015) documented that, intracellularly, SNAP47 regulates the localization and function of a subset of vesicle-associated membrane proteins (VAMPs). Epitope-tagged SNAP47 localized partially to endosomes and predominantly to the endoplasmic reticulum (ER) and ER-Golgi intermediate compartment (ERGIC), as SNAP47 interacted with syntaxin-5 (STX5) and syntaxin-1 (STX1) when retained in the ER (Kuster et al. 2015). SNAP47 has also been shown to cooperate with VAMP5, STX1B, and STX4 in the regulation of exocytosis (Matsui et al. 2023). As a genuine SNARE, SNAP47 is also likely to be involved in SNARE complex formation during intracellular fusion reactions. In fact, recently, Jian et al. (2024) provided evidence demonstrating that SNAP47 is primarily localized to mitophagosomes and forms a ternary SNARE complex with autophagosomal STX17 and lysosomal VAMP7/VAMP8 to mediate autophagosome–lysosome fusion.

At the cellular level, SNAP47 is trafficked to postsynaptic compartments and appears to be involved in postsynaptic fusion events in hippocampal glutamatergic neurons. Jurado et al. (2013) identified a critical role for SNAP47, together with STX3 and synaptobrevin-2, as components of a unique postsynaptic SNARE-mediated vesicle fusion machinery required for delivery of the α-amino-3-hydroxy-5-methyl-4-isoxazolepropionic acid receptors (AMPARs) to the plasma membrane during long-term potentiation (LTP). Endogenous SNAP47 is distributed to axons in cortical neurons, but appears to be unnecessary for neurotransmission, exocytosis, and the recycling of SVs. However, it appears to regulate the release of brain-derived neurotrophic factor (BDNF) and positively modulate the layer-specific branching of callosal axons in pyramidal neurons in the somatosensory cortex in vivo (Shimojo et al. 2015).

Most existing studies of SNAP47 function have generally focused on intracellular processes, but have not precisely distinguished between different types of neurons in defined brain areas. In our previous work (Münster-Wandowski et al. 2017), we found divergent distribution patterns of SNAP47 in the mouse and rat hippocampus. While SNAP47 was broadly distributed in all areas and layers of the hippocampus in both mouse and rat, in the mouse, the strongest expression was observed in VGAT-positive inhibitory INs. In the rat, the strongest expression was in the termination zone of the excitatory mossy fiber projection. At the subcellular level, SNAP47 localized to both postsynaptic and presynaptic elements, with higher density in postsynaptic elements in both mouse and rat. We demonstrated that GABAergic neurons in the mouse hippocampus synthesize SNAP47 at high levels and that the functional protein can be localized both postsynaptically in somato-dendritic compartments and presynaptically in inhibitory axon terminals. This suggests that SNAP47 may play a role in inhibitory transmission by participating not only in postsynaptic events but also in neurotransmitter release at presynaptic sites.

In this study we investigated whether knocking-down the SNAP47 protein affects the morphology and transmission of hippocampal neurons, with a particular focus on GABAergic inhibitory neurons (Münster-Wandowski et al. 2017; Holt et al. 2006). To address this, we developed a lentiviral vector carrying a shRNA to knockdown SNAP47. Immunocytochemical and electrophysiological experiments were performed on autaptic neuronal cultures of the hippocampus from transgenic mice that express YFP-Venus selectively in GABAergic INs under the control of the VGAT promoter, enabling their identification and targeting.

## Results

### Development of a Viral-Mediated Knockdown of SNAP47

To evaluate the function of SNAP47, we constructed a lentiviral-based SNAP47 shRNA knockdown to silence the protein level in individual hippocampal neurons and determine whether the absence of SNAP47 affects the morphology and neurotransmission of the hippocampal inhibitory GABAergic neurons.

**Fig. 1:**
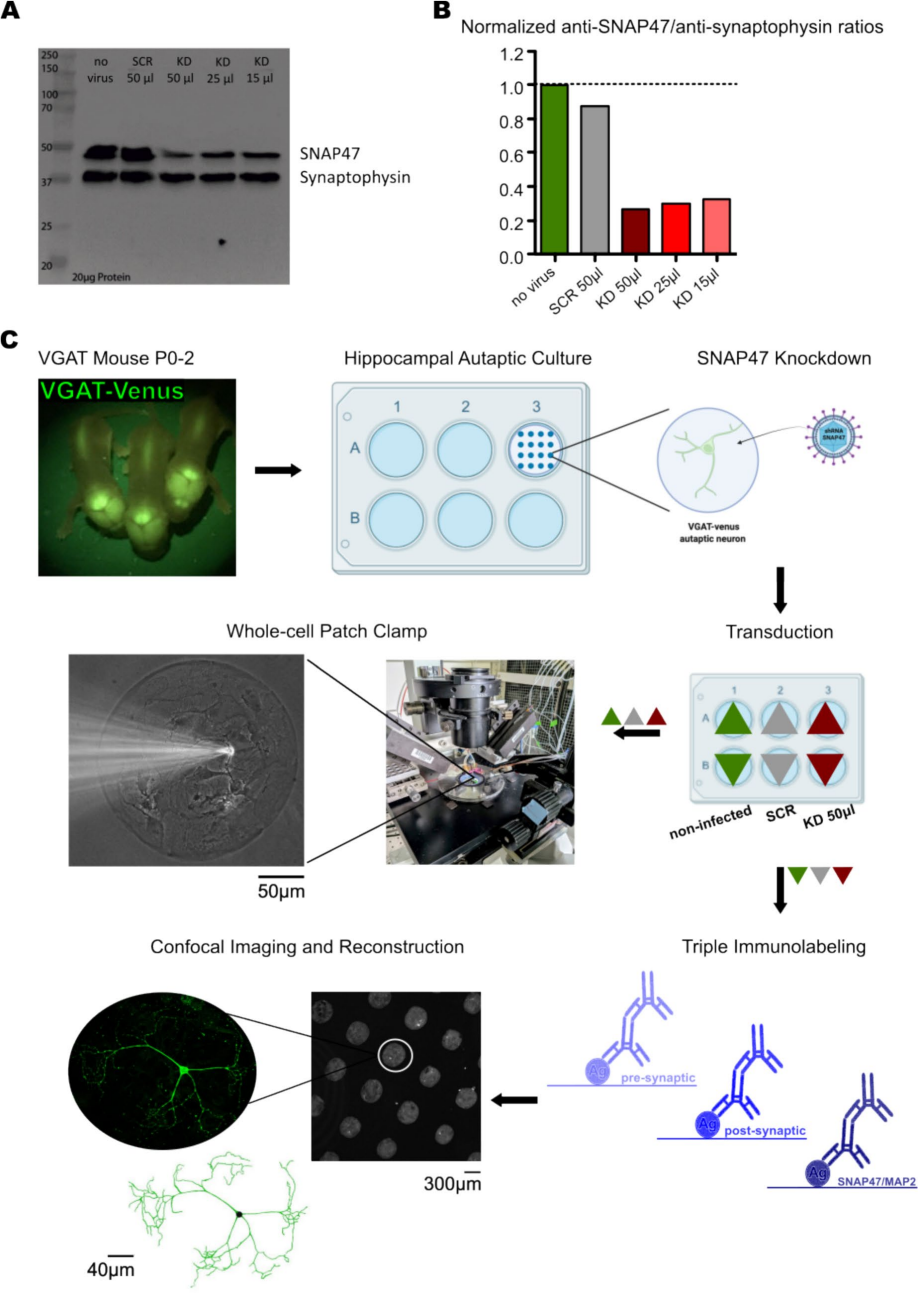
The efficiency of a lentivirus shRNA knockdown of the SNAP47 protein. **A** Western blot of high-density hippocampal cultures non-infected (no virus), treated with scrambled shRNA (SCR) and different amounts of SNAP47-shRNA (KD) lentivirus. **B** Quantification shows reduced up to ~ 80% expression of SNAP47-shRNA virus (50 µl) in the KD neurons relative to no virus controls and those transfected with a scrambled vector. **C** The experimental workflow showing the preparation of the hippocampal autaptic cultures prepared from VGAT-Venus mice (P0—2), followed by the transduction of lentivirus shRNA SNAP47 KD and SCR. The hippocampal neurons were ready for electrophysiology and immunocytochemistry at DIV14 after infection. Created using the Inkscape 1.2 graphic program

Initially, we generated two shRNA lentivirus, one which express shRNA (SNAP47-shRNA) to knockdown mouse SNAP47 protein and a control virus expressing a scrambled shRNA sequence of the clathrin protein (SCR-shRNA). Both shRNAs were fused to a P2A linker after a nuclear localized (NL) RFP sequence (NLS-RFP), in order to express the enhanced red fluorescent protein (RFP) for the detection of transfected neurons. We first, validated the efficiency of our SNAP47 knockdown approach by Western blot. High density hippocampal neuronal cultures from C57BL/6 mice were treated with increasing amounts of SNAP47-shRNA (KD) lentivirus: 15, 25 and 50 µl and compared with 50 µl of control virus scramble-shRNA (SCR) treated and with non-infected hippocampal cultures (i.e. no virus applied) (Fig. 1A). We selected synaptophysin as a control housekeeping protein, as it is expressed in all types of hippocampal neurons and we quantified the SNAP47 signal normalized to the synaptophysin band. We observed a reduction of SNAP47 protein expression with up to the ~ 80% decrease when we used 50 µl of the shRNA KD when compared with the SCR shRNA treated and non-infected cultures (Fig. 1B).

We next used the 50 µl shRNA KD virus to validate the degree of infection of the generated viruses using the NLS-RFP signal in high density hippocampal cultures neurons. 50 µl of virus that correspond to 3,75 X 10^6^ infected units (IU)/ml of SCR-shRNA or 3,60 X 10^6^ IU/ml of SNAP47-shRNA lentiviruses, were added onto the hippocampal neuronal cultures at DIV 1 and the nuclear RFP expression was observed and quantified at DIV 15 with a fluorescence microscope (Supplementary-Fig. 1). Both lentiviruses showed no cytotoxic effect as we did not observe any evident difference in cell death between cultures infected with the lentivirus expressing SCR-shRNA and SNAP47-KD, and the non-infected cultures. Quantification of the RFP fluorescent positives nucleus in these cultures revealed that all viruses reached up to a 100% infection efficiency whether expressing the shRNA knockdown (SNAP47-KD) or the scrambled sequence (SNAP47-SCR) (Supplementary-Fig. 1).

These data indicate that our knockdown approach efficiently reduces the endogenous SNAP47 protein expression and infects the majority of the neurons in hippocampal cultures.

In view of the high infection efficiency and lack of obvious toxic effects, we used 50 µl virus in our subsequent experiments. The experimental design for using lentivirus expressing SNAP47-shRNA in single hippocampal neurons and distinguishing between GABAergic and glutamatergic neurons is shown schematically in Fig. 1C. We cultured autaptic hippocampal neurons from postnatal day 0—2 (P0—2) VGAT-YFP-positive mice according to the methodology described by Arancillo et al. (2013). These cultures allowed us to grow neurons individually on astrocytic microislands. Using YFP labeling, we could accurately distinguish between GABAergic inhibitory neurons (VGAT-YFP-positive) and glutamatergic excitatory neurons (VGAT-YFP-negative) within the cultures. We then administered 50 µl of SCR-shRNA and SNAP47-KD lentivirus to infect neurons 24 h after plating in two separate sets of autaptic cultures. Fourteen days after transduction, one set of autaptic hippocampal neurons was fixed for immunocytochemical analysis to evaluate the effects of SNAP47-KD on morphology, while another set was used for whole-cell patch-clamp recordings to evaluate the effect on neurotransmission.

### SNAP47 Knockdown Affects the Structural Organization of Hippocampal Inhibitory GABAergic Neurons

**Fig. 2:**
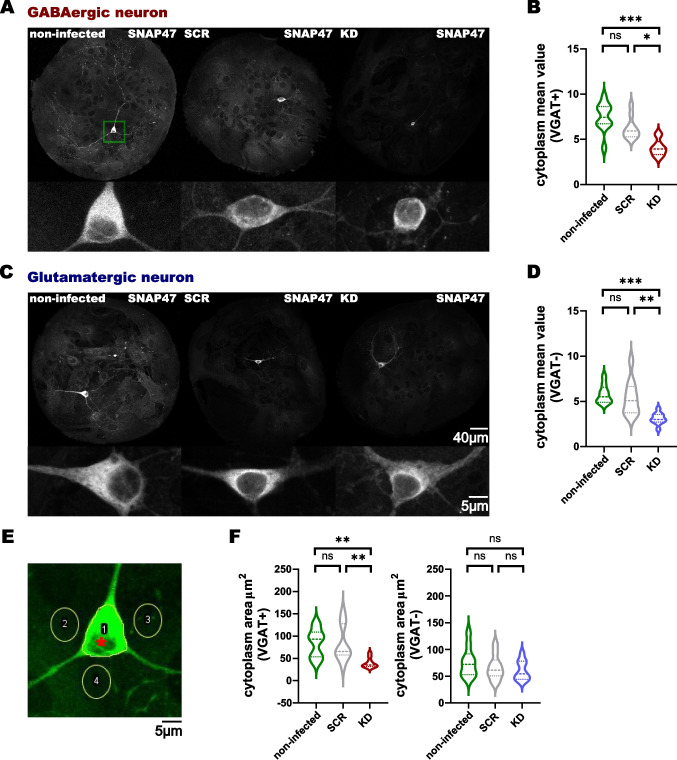
Quantitative labeling of SNAP47 protein in GABAergic and glutamatergic autaptic cultured neurons. **A**, **C** Representative images of astrocytic islands and magnified views of the soma and dendrites showing the intensity of the SNAP47 signal in both GABAergic and glutamatergic autaptic neurons. **B**, **D** The quantification of the mean intensity SNAP47 signal of cell body excluding nucleus in 3 different groups. (B *p* = 0.0001 between non-infected and KD, *p* = 0.0248 between SCR and KD, *n* = 10 for each group; D *p* = 0.0004 between non-infected and KD, *p* = 0.0045 between SCR and KD, *n* = 10 for each group). **E** An illustration of the neuron soma from (**A**) showing the signal-to-noise ratio calculation for (**B** and **D**), where the intensity of region 1 (* indicates the nucleus, which is excluded) is divided by the average intensity of region 2, 3, and 4. **F** The quantification of the size of the cell body excluding nucleus. VGAT + : *p* = 0.0041 between non-infected and KD, *p* = 0.0047 between SCR and KD, *n* = 10 for each group)

To confirm the reduction of endogenous SNAP47 in individual hippocampal neurons, we prepared autaptic neuronal cultures from P0—2 VGAT-YFP mice and SNAP47 levels in GABAergic or glutamatergic neurons were assessed by immunocytochemistry. We included SCR-shRNA-treated and non-infected hippocampal autaptic cultures as controls. 14-day post-infection, we fixed autaptic cultures from VGAT-YFP mice infected with the SCR-shRNA and SNAP47-KD lentivirus, as well as non-infected cultures, and immunostained them for SNAP47. We analyzed 10 islands populated with a single VGAT-YFP-positive GABAergic and 10 with VGAT-YFP-negative glutamatergic neurons (Fig. 2A, C). We have quantified the somatic immunolabeling intensity signal for SNAP47 for each group (non-infected, SCR and KD) and normalized it to the background, which in this case was the surrounding glial cells which form the microisland (Fig. 2E). We chose to measure only the somatic signal of this protein, because we know from our previous work (Münster-Wandowski et al. 2017) that SNAP47 is massively somatically accumulated in the majority of GABAergic neurons, at much higher levels than in the neuronal processes. In the three experimental groups (non-infected, SCR, and KD), therefore, we measured the mean intensity (arbitrary gray scale units) of SNAP47 labeling intensity in the somatic cytoplasm (excluding not stained nucleus) of VGAT-YFP-positive GABAergic and VGAT-YFP-negative glutamatergic cell bodies of autaptic hippocampal neurons. In hippocampal neurons-treated with SNAP47-KD lentivirus, we observed a reduction of the SNAP47 signal in both neuronal types compared to infected autaptic hippocampal neurons-treated with scrambled lentivirus and non-infected single neurons. In KD-treated GABAergic VGAT-YFP-positive neurons, the mean intensity was reduced to an average of 65% in the KD group (4.079 ± 0.2864 Gy scale value, Fig. 2B) compared to the mean cytoplasm/background ratio of SCR (6.230 ± 0.3829) and non-infected (7.453 ± 0.5047). When the mean cytoplasmic intensity of glutamatergic VGAT-YFP-negative cell bodies in autaptic hippocampal neurons was assessed, the SNAP47 intensity in KD-treated neurons was reduced to an average of 57%: 3.106 ± 0.1994 Gy scale value (Fig. 2D), compared to a mean cytoplasm/background ratio of 5.406 ± 0.6046 in SCR and 5.799 ± 0.3420 in non-infected neurons.

Overall, the SNAP47 showed significantly reduced somatic intensity of SNAP47 in KD-treated neurons in both types of neurons, VGAT-YFP-positive and VGAT-YFP-negative, validating the efficiency of the lentiviral vector at the single cell level.

In addition, during the SNAP47 somatic labeling analysis we noticed a reduced size of neuronal somas in VGAT-YFP-positive GABAergic neurons compared to the KD-treated VGAT-YFP-negative glutamatergic neurons (Fig. 2A–C). We used the signal from the previous immunolabeling of SNAP47 to quantify the cytoplasmic area in the soma in both cell types. We compared the cytoplasmic area of the neuronal body (excluding the nucleus) of VGAT-YFP-positive and YFP-negative neurons in each group (non-infected, SCR and KD) using a one-way ANOVA and semi-automated tracing in ImageJ (Fig. 2E). We found that the total cytoplasmic area was significantly smaller in the KD VGAT-YFP-positive INs (39.43 ± 3.452 µm^2^) compared to the SCR VGAT-YFP-positive group (83.09 ± 12.81 µm^2^, *p* = 0.0047 between KD and SCR) and non-infected VGAT-YFP-positive INs (84.79 ± 10.01 µm^2^, *p* = 0.0041 between KD and non-infected; Fig. 2F). The size of the soma was similar in the two control groups, SCR and non-infected (*p* > 0.9999 between SCR and non-infected).

In contrast, KD VGAT-YFP-negative glutamatergic neurons show no significant differences in soma size (60.81 ± 5.779 µm^2^, *p* > 0.9999 between KD and SCR), and did not differ between the SCR (66.06 ± 7.260 µm^2^) and non-infected (76.10 ± 8.642 µm^2^, *p* = 0.4870 between KD and non-infected, *p* > 0.9999 between SCR and non-infected; Fig. 2F).

These results suggest that reducing the amount of SNAP47 protein in GABAergic, VGAT-YFP-positive neurons significantly decreases the area of somatic SNAP47.

### SNAP47 Knockdown Alters the Dendritic Development of Hippocampal Inhibitory GABAergic Neurons

Using SNAP47 labeling, we observed that autaptic neurons exhibited typical features of differentiated neurons in culture, such as long projections, including extensive axons and dendritic trees. However, visual inspection of VGAT-YFP-positive GABAergic neurons suggested that their dendritic trees were shorter following infection with SNAP47 shRNA. Given the anticipated weak and significantly reduced SNAP47 signal in this group, accurate analysis of these neuronal processes was challenging. Therefore, to validate our observations and confirm the changes to the neuronal morphology of this group, we employed MAP2 labeling to conduct a more reliable analysis. We analyzed the dendritic morphology of inhibitory VGAT-YFP-positive and excitatory VGAT-YFP-negative autaptic hippocampal neurons using MAP2 (Microtubule-associated protein 2) immunocytochemistry and semi-automated tracing (ImageJ) to reconstruct neuronal processes.

**Fig. 3:**
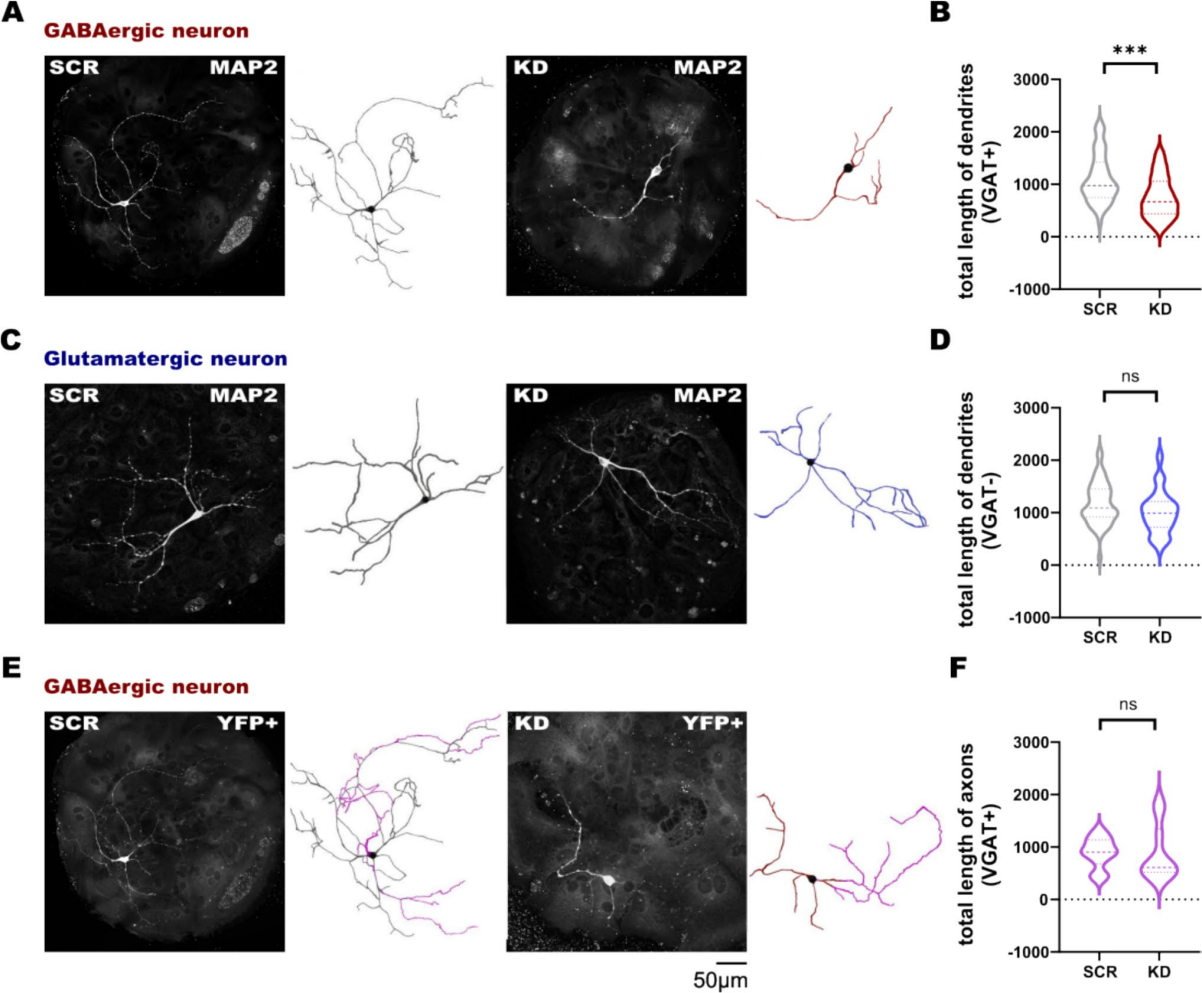
The dendritic and axonal morphology of GABAergic and glutamatergic autaptic cultured neurons. **A**,** C** The MAP2 labeling and the dendrites reconstruction of GABAergic neuron and glutamatergic neuron in 2 treated groups—SCR and KD. **B**,** D** The quantification of the total length of dendrites (B *p* = 0.0001, n(SCR) = 49, n(KD) = 46; D n(SCR) = 47, n(KD) = 45). **E**,** F** The YFP + labeling and the axonal morphology in magenta and the quantification of the total length of axons in GABAergic neurons (*n* = 10 for each group)

Quantitative analysis of MAP2-positive neurites showed that the mean total dendritic length of VGAT-YFP-positive GABAergic neurons in the KD group was significantly reduced, measuring 755.9 ± 58.23 µm, compared to the SCR (1141 ± 70.08 µm, *p* = 0.0005 between KD and SCR) and non-infected (1562 ± 185.9 µm, *p* = 0.0001 between KD and non-infected) groups (Fig. 3A, B, Supplementary Fig. 2A). In comparison, the total dendritic length of VGAT-YFP-negative glutamatergic neurons in the KD-treated group (1009 ± 61.31 µm) was significantly not affected compared to the control scrambled (1366 ± 71.55 µm) and non-infected (1600 ± 111.5 µm) groups (Fig. 3C, D, Supplementary Fig. 2B).

As the total dendritic length was significantly reduced in GABAergic KD neurons but remained unchanged in glutamatergic neurons, we only measured axon length in the former group based on transgenic YFP expression. Axon length was quantified exclusively in GABAergic neurons identified by transgenic YFP expression, as glutamatergic neurons lacked YFP expression and therefore could not be reliably distinguished for this measurement. The semi-automated analysis of the axonal length in the GABAergic VGAT-YFP-positive neurons did not show any significant differences between the KD group (875.7 ± 165.7 µm) and the SCR group (896.4 ± 91.68 µm, *p* = 0.6305 between both) and between SCR and non-infected group (838 ± 83.70 µm, *p* = 0.5787 between both; Fig. 3E, F, Supplementary Fig. 2A).

These results demonstrate that the SNAP47 protein deficit is reflected in the morphology of GABAergic hippocampal neurons only, resulting in a significant reduction in total dendritic length with invariant axon length.

### SNAP47 Knockdown Reduces Pre- and Postsynaptic Marker Expression in Hippocampal Inhibitory GABAergic Neurons

The significant reduction in the size of the dendritic trees observed in GABAergic neurons in KD using MAP2 immunolabeling raises another question: what potential effects might this structural alteration have on other anatomical characteristics, in particular, the synaptic connectivity of the GABAergic hippocampal neuron?

**Fig. 4:**
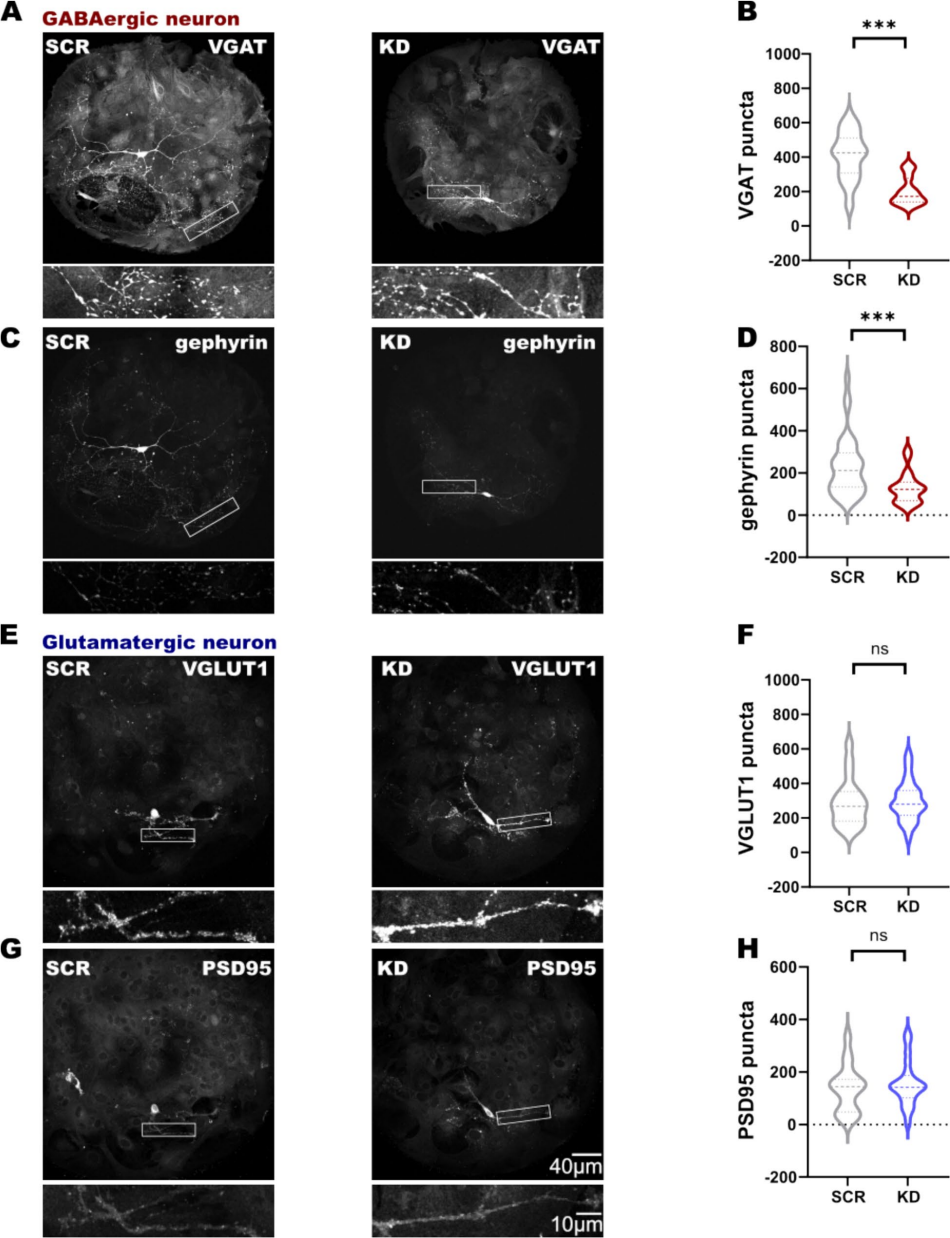
Double immunolabeling for pre- and postsynaptic markers in GABAergic and glutamatergic autaptic cultured neurons. **A–D** Double immunolabeling for pre- and postsynaptic GABAergic markers (VGAT and gephyrin, respectively) and a close-up image of the puncta on dendrites for each neuron in two treated groups: SCR and KD. Note the significant reduction of VGAT and gephyrin signal in KD neurons. (B *p* < 0.0001 between SCR and KD, n(SCR) = 35, n(KD) = 31; D *p* = 0.0002 between SCR and KD, n(SCR) = 35, n(KD) = 31). **E–H** Double immunolabeling for pre- and postsynaptic glutamatergic markers (VGLUT1 and PSD95, respectively) and a close-up image of the puncta on dendrites for each neuron in two treated groups: SCR and KD. The quantification shows no difference on the pre- and postsynaptic markers in 2 treated groups (F n(SCR) = 18, n(KD) = 28; H n(SCR) = 16, n(KD) = 21)

To answer this question, we first decided to perform a series of double immunolabeling with pre- and postsynaptic markers to identify synapses in GABA- and glutamatergic hippocampal neurons (VGAT/gephyrin, VGLUT1/PSD95, respectively) in the three groups: SNAP47-KD, scrambled and non-infected (Fig. 4, Supplementary-Fig. 2C-D).

The quantified total number of the VGAT puncta (the vesicular inhibitory amino acid transporter) in the presynaptic compartment and gephyrin puncta (a central protein that anchors, clusters and stabilizes glycine and γ-aminobutyric acid type A receptors at inhibitory synapses) in the postsynaptic compartment in KD and SCR GABAergic VGAT-YFP-positive neurons were: 201.4 ± 14.33 for VGAT and 127.2 ± 12.9 for gephyrin in the KD group, and 409.2 ± 23.32 for VGAT and 218.9 ± 20.73 gephyrin in the SCR group, respectively (Fig. 4A–D). Thus, we observed a strong reduction of VGAT and gephyrin puncta in SNAP47-KD GABAergic INs (VGAT: *p* < 0.0001 and gephyrin: *p* = 0.0002 between KD and SCR; Mann–Whitney, non-parametric test; Fig. 4B and D). There was no significant difference in VGAT and gephyrin puncta between SCR (409.2 ± 23.32 for VGAT and 218.9 ± 20.73 for gephyrin) and non-infected group (504.6 ± 56.11 for VGAT and 309 ± 41.29 for gephyrin; Supplementary-Fig. 2C).

In contrast, the quantification in glutamatergic VGAT-YFP-negative neurons didn’t reveal any differences. The total number of the presynaptic VGLUT1 puncta (the vesicular excitatory glutamate transporter 1) and postsynaptic PSD95 (the postsynaptic density protein 95, the postsynaptic scaffolding protein in excitatory neurons) puncta in SNAP47-KD was: 299.3 ± 21.94 and 154.9 ± 18.61, respectively, and in SCR 290.4 ± 32.8 and 137.8 ± 22.24, respectively (Fig. 4E-H), indicating no reduction in VGLUT1 and PSD95 puncta in SNAP47-KD glutamatergic neurons. Similarly, there were no significant differences between SCR and non-infected groups (322.3 ± 36.12 and 172.9 ± 21.42, respectively; Supplementary Fig. 2D) in glutamatergic neurons.

In conclusion, knocking down SNAP47 led to a substantial decrease in the number of VGAT and gephyrin puncta in GABAergic neurons, indicating a possible decrease in synaptic connections. In contrast, the total number of pre- and postsynaptic markers remained unchanged in glutamatergic neurons. This suggests that the previously observed reduction in dendritic length, restricted to GABAergic neurons, may underlie the impairment of inhibitory synapses.

### SNAP47 Knockdown Decreases Inhibitory GABAergic Transmission

The observation that SNAP47 knockdown showed affected cell morphology, such as reduced somatic cytoplasmic area, dendritic length, and total number of pre- and postsynaptic puncta in GABAergic VGAT-YFP-positive neurons, raise the possibility of an impairment of GABAergic synaptic transmission. Therefore, we next investigated the effects of SNAP47 knockdown on inhibitory synaptic transmission in autaptic hippocampal neurons from VGAT-YFP-positive mice.

Initially, to discard potential experimental artifacts arising from the use of lentivirus, we examined synaptic transmission by measuring spontaneous and Ca^2+^ evoked neurotransmitter release in inhibitory autaptic neurons transduced with shRNA SCR compared to non-infected autaptic neurons. We examined the miniature inhibitory postsynaptic current (mIPSC) frequency that can suggest alteration from either the presynaptic or postsynaptic site and mIPSC amplitudes that can show changes at the postsynaptic site. Our findings revealed similar normalized mean values for mIPSC frequencies (non-infected 1.00 ± 0.10 and SCR 0.856 ± 0.09) as well as for the amplitudes (non-infected 1.00 ± 0.07 and SCR 1.07 ± 0.10) of mIPSCs (Supplementary-Fig. 3A-B). We also assessed Ca^2+−^evoked release by measuring the amplitudes and charge of Ca^2+^-evoked inhibitory postsynaptic currents (IPSCs), in individual control neurons expressing the reporter NLS-RFP SCR, compared with the non-infected neurons. The normalized IPSC amplitudes and charges (Supplementary-Fig. 3C-D) showed comparable mean values in both groups; amplitudes (non-infected 1.00 ± 0.11 and SCR 1.28 ± 0.16) and charges (non-infected 1.00 ± 0.14 and SCR 0.88 ± 0.15). Confirming, that the use of lentivirus does not affect the inhibitory synaptic transmission. As expected, the use of lentivirus also had no effect on excitatory synaptic transmission. The normalized mean miniature excitatory postsynaptic current (mEPSC) frequency and amplitude, as well as the Ca^2+^-evoked excitatory postsynaptic current (EPSC) amplitude and charge values (see Supplementary Fig. 3E–H), were similar in the non-infected and SCR groups.

**Fig. 5:**
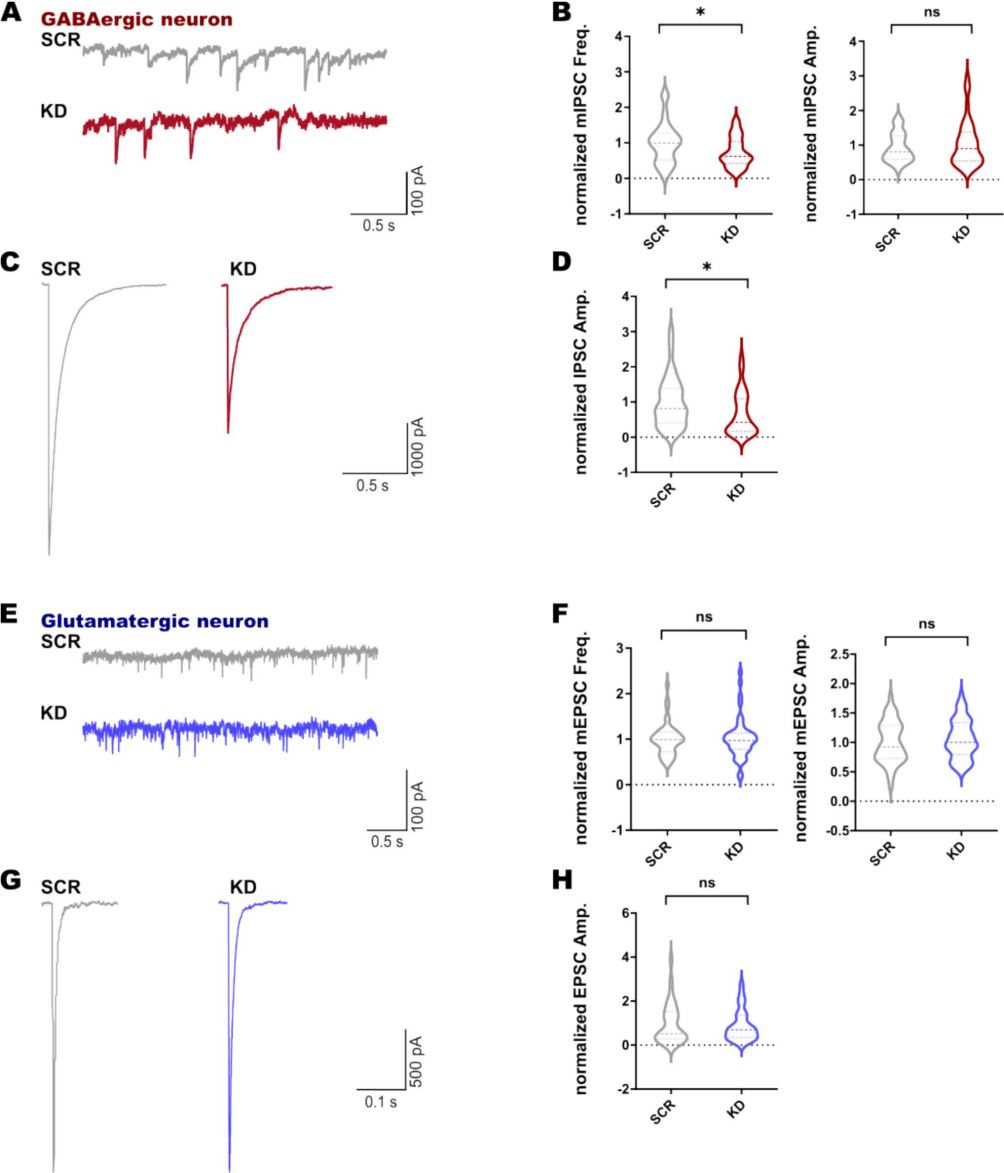
Electrophysiological characterization of GABAergic and glutamatergic synaptic transmission in an autaptic cell culture system. **A**, **B** Representative recording traces of mIPSC and the quantification of the normalized frequency and amplitude in SCR and KD (*p* = 0.0363, n(SCR) = 42, n(KD) = 41). **C**, **D** Representative recording traces of evoked IPSC and their normalized amplitude measurements in SCR and KD (*p* = 0.0167, n(SCR) = 42, n(KD) = 41). **E**, **F** Representative recordings of mEPSC and the quantification of the normalized frequency and amplitude in SCR and KD (n(SCR) = 43, n(KD) = 47). **G**, **H** Representative recordings of evoked EPSC and their normalized amplitude measurements in SCR and KD (n(SCR) = 46, n(KD) = 46)

Next, we focused on the impact of reduced SNAP47 expression in synaptic transmission in VGAT-YFP-positive hippocampal GABAergic inhibitory autaptic neurons transduced by SNAP47-KD lentivirus. We first assessed the effects on spontaneous release and observed a notable significant decrease of about 30% in the frequency of mIPSCs recorded from autaptic VGAT-YFP-positive hippocampal GABAergic inhibitory neurons KD compared with SCR controls (Fig. 5A and B). These changes could arise from influences at either the presynaptic or postsynaptic level. To dissect which aspect was predominantly affected, we analyzed the amplitude of mIPSCs (Fig. 5B) and found no significant difference in the mean normalized mIPSC amplitudes between the KD and control groups suggesting that this effect likely originates from the presynaptic level.

We also recorded IPSC induced by a single action potential (AP) to address the impact of reducing SNAP47 expression levels on Ca^2+^-evoked release in autaptic hippocampal neurons. We analyzed the amplitude of AP-evoked IPSCs and revealed that knockdown of SNAP47 also impaired Ca^2+^-triggered release (Fig. 5C and D). We observed that amplitudes of IPSCs were a 36% smaller in SNAP47-KD neurons compared to the control SCR neurons (Fig. 5D).

To investigate whether excitatory neurotransmission was also affected by the silencing of SNAP47, we performed the same analysis in VGAT-YFP-negative glutamatergic autaptic neurons infected with shRNA SNAP47-KD or control shRNA scrambled lentivirus (Fig. 5E–H). We found no differences in spontaneous release, as indicated by the analysis of frequencies and amplitudes of the mEPSCs recorded (Fig. 5E and F). Similarly, Ca^2+^-evoked release, assessed by the EPSC analysis, remained unaffected in VGAT-YFP-negative neurons (Fig. 5G and H).

Taken together, these results suggest that SNAP47 deficiency significantly impacts inhibitory GABAergic neurotransmission in hippocampal neurons, while having no effect on glutamatergic transmission.

## Material and Methods

### Animals

VGAT-Venus transgenic mice have been described previously (Wang et al. 2009). In these transgenic animals Venus is expressed highly selectively in 95—98% of cortical GABAergic neurons in the neocortex and the hippocampus. VGAT-Venus transgenic mice and rats exhibit otherwise normal growth and reproductive behavior. The VGAT-Venus animals were crossed and the postnatal (P) day 0—2 offspring of comparable weight were used. The sex of the P0—2 animals used for experimentation was not distinguished.

All procedures and animal maintenance were performed in accordance with the relevant Institutional guidelines and regulations, the National Act on the Use of Experimental Animals (Germany), local authorities (LaGeSo: Regional Office for Health and Social Affairs in Berlin, registration numbers: Reg 0254/19, O-0098/12, T-CH 0040/20 and Animal Welfare Committee of Charité-Universitätsmedizin Berlin: license no. T 0220/09) and the current European Council Directive 2010/63/EU.

### Lentiviral Constructs and Virus Production

The lentiviral constructs were based on the lentiviral shuttle vector FUGW (Lois et al. 2002) and contained a human U6-controlled shRNA cassette in addition to the human synapsin-1 promoter-driven nuclear localized (NLS) RFP expression cassette. A mouse SNAP47 specific shRNA (ATAGCAATAGAATCAGCAGAGC) previously validated by Jurado et al. (2013) linked via TTCAAGAGA was cloned in the multiple cloning site (MCS). As control served the same vector harboring a scrambled shRNA against rat clathrin (Watanabe et al. 2014).

Lentiviral particles were prepared by the Charité Viral Core Facility (https://vcf.charite.de/) as previously described (Lois et al. 2002; vcf.charite.de). Briefly, HEK293T cells were co-transfected with the shuttle vector f(U6) SNAP47-shRNA-Syn-NLS.RFP-WPRE and helper plasmids, pCMVdR8.9 and pVSV.G with polyethylenimine. Virus containing supernatant was collected after 72 h, filtered, aliquoted, flash-frozen with liquid nitrogen, and stored at—80 °C. In order to determine the effective titer of the preparation, 10^5^ hippocampal neurons per well in a 6 well plate were infected with different volumes (10—50 µl) of lentivirus. After 14 DIV, the ratio of infected (visible by NLS-RFP fluorescens) versus non-infected cells was determined through visual inspections and cell counting in three fields of views. The infection units per ml, were calculated by multiplying this ratio by the number of cells seeded (100 K) and divided by the amount of virus used (in ml).

### Detection of Protein Expression by Western Blotting

In order to determine whether the SNAP47-shRNA lentivirus reduced SNAP47 protein expression, the levels of SNAP47 in infected and control cells were compared.

For quantification of SNAP47 protein levels, hippocampal neurons were lysed 14 days post transduction at 4 °C with lysis buffer (50 mM Tris, pH 8.0, 150 mM NaCl, 0.2% NP-40, protease inhibitor cocktail complete mini; Roche Diagnostics). Equal amounts of total protein from the lysates of SNAP47-KD, scrambled control virus and non-infected were separated on SDS polyacrylamide gel and transferred to a nitrocellulose membrane. Membranes were blocked for 1 h with 5% skim milk in PBS-T and incubated at 4 °C overnight with primary antibodies: anti-SNAP47 (111,403 Synaptic Systems) and anti-Synaptophysin (101,011 Synaptic System). Secondary antibodies were horseradish peroxidase-conjugated (Jackson ImmunoResearch, Cambridgeshire, UK). Detection was performed by using ECL Plus Western Blotting Detection Reagents (GE Healthcare Biosciences) in a Fusion FX7 detection system (Vilber Lourmat). Data were analyzed offline using ImageJ.

### High-Density Hippocampal Neuronal Cultures, Autaptic Hippocampal Neuronal Culture, and Viral Infection

To evaluate the efficiency of our knockdown approach and viral infection, we established high-density hippocampal cultures derived from C57Bl6 mice at postnatal days (P) 0–2. Following previously established protocols (Arancillo et al. 2013), cortical astrocyte feeder layers were prepared 1–2 weeks prior to hippocampal neuronal culture. Hippocampal cultures were prepared from mice of both sexes. Neuronal seeding onto the astrocytes was conducted at densities: 100,000 cells per well for lentiviral titer determination (Supplementary-Fig. 1) and for western blot analysis (Fig. 1).

For immunocytochemistry experiments and electrophysiology recordings, autaptic GABAergic- and glutamatergic hippocampal neurons were cultured. Autaptic hippocampal neurons were prepared as described previously (Arancillo et al. 2013). Briefly, astrocytes were obtained from cerebral cortices of postnatal day (P0–2) C57BL/6N mice and plated at a density of 5000 cells/cm2 on a PDL/collagen micropattern coverslips 1 week before of the preparation of the neurons. Hippocampal neurons derived from VGAT-Venus postnatal (P0—2) mice of either sex were obtained after enzymatic digestion with papain solution (Worthington). Neurons were plated at low density of about 3000 cells on 30 mm coverslips containing the astrocytic microislands. Either high density or autaptic hippocampal cultures were infected with the appropriate viral construct (about 1.8 × 10^5^ IU) 24 h after plating and maintained at 37 °C and 5% CO2 for 14 days.

Although less commonly used than conventional neuronal cultures, the autaptic system offers several advantages relevant to this study. In this model, a single neuron is grown on a microisland of astroglia, forming synapses exclusively with its own dendrites, which allows quantitative assessment of both morphological and functional properties. It is particularly suited for molecular manipulations, such as viral-mediated shRNA delivery, since changes in the recorded cell can be directly linked to presynaptic or postsynaptic protein alterations. Functionally, the system enables precise measurement of evoked and spontaneous neurotransmitter release, as well as the effects of pharmacological agents. Morphological analyses are confined to a single neuron, free from network influences, greatly simplifying the interpretation of structural changes compared to conventional cultures.

### Immunofluorescence Labeling

Autaptic hippocampal neurons were fixed with buffered 4% paraformaldehyde (PFA; Sigma-Aldrich) for 10 min at DIV14 after transduction, blocked and incubated with a mixture of primary antibodies. The single-labeling antibody was for SNAP47 and MAP2. Double immunostaining for SNAP47 and several specific GABA- and glutamatergic pre- and postsynaptic markers were used to determine the compartmental distribution of the SNAP47 protein in autaptic GABA- and glutamatergic hippocampal neurons. Double-labeling antibody combinations were as follows: SNAP47 + VGAT, SNAP47 + gephyrin, SNAP47 + VGLUT1, SNAP47 + PSD95. For detection of the primary antibodies autaptic hippocampal cultures were subsequently incubated in a mixture of the appropriate secondary antibodies: goat anti-mouse (1:300), goat anti-rabbit (1:500), goat anti-guinea pig (1:500) conjugated to Alexa Fluor series fluorochromes (Alexa Fluor 647 anti-mouse, Alexa Fluor 488 anti-rabbit, Alexa Fluor 405 anti-guinea pig). For a comprehensive list of the antibodies including their characteristics, dilution and source please see Table 1. After immunofluorescence labeling autaptic hippocampal cultures were subsequently mounted in Fluorsave mounting medium (Calbiochem, San Diego, CA) coverslipped and examined using a confocal microscope (FV1000, Olympus, Hamburg, Germany).

**Table 1:** List of primary antibodies for immunocytochemical analysis to evaluate the effects of SNAP47-KD on morphology

Antibody	Supplier and Cat. No	Host	Dilution	Immunogen
SNARE protein				
SNAP47	Synaptic System 111 403	Rabbit (polyclonal, affinity purified)	1:300	Recombinant full-length rat SNAP47
Pre-synaptic GABA- and glutamatergic marker protein				
VGAT	Synaptic System 131 004	Guinea pig (polyclonal antiserum)	1:200	Recombinant protein corresponding to residues near the amino terminus of rat VGAT. K.O. validated
VGAT	Synaptic System 131 011	Mouse (monoclonal purified IgG)	1:200	Synthetic peptide corresponding to residues near the amino terminus of rat VGAT
VGLUT1	Synaptic System 135 304	Guinea pig (polyclonal, crude antiserum)	1:2000	Purified recombinant protein of rat VGLUT1 (aa 456—560)
Post-synaptic GABA- and glutamatergic marker protein				
gephyrin	Synaptic System 147 111	Mouse (monoclonal purified IgG)	1:100	Recombinant protein corresponding to AA 307 to 735 from rat Gephyrin
PSD95	UC Davis/NIH NeuroMab Facility 75—028	Clone K28/43 Mouse (monoclonal)	1:100	Fusion protein amino acids 77—299 (PDZ domains 1 and 2 of human PSD95)
Neuron-specific cytoskeletal proteins enriched in dendrites and perikarya				
MAP2	Millipore MAB 3418	Purified mouse (monoclonal IgG1)	1:100	Bovine brain microtubule protein
MAP2	SYSY 188,004	Guinea pig (polyclonal antiserum)	1:1000	Recombinant protein corresponding to residues near the amino terminus of human Map2

### Confocal Imaging and Quantitative Analysis

For quantitative assessment, all groups (non-infected, scrambled, and knockdown) compared in an experiment were processed in parallel using identical antibody solutions and other reagents. Three (for SNAP47 and MAP2) and five (for pre- and postsynaptic protein puncta) independent cultures per group (non-infected, scrambled, and knockdown) were imaged and analyzed for each experiment.

In order to get an overview of SNAP47 expression and the morphology of the GABA- and glutamatergic hippocampal individual neuron we imaged the autaptic neuronal cultures using an × 4 objective lens on a confocal laser-scanning microscope (Olympus FV1000) and arranged overview images (showed in Fig. 1A). Higher-resolution images were acquired using an × 30 silicon oil immersion lens for SNAP47 signal intensity (as showed in Fig. 2A, C), × 20 objective lens for MAP2 and YFP (as showed in Fig. 3A, C, E) to resolve individual pre- and postsynaptic puncta (as showed in Fig. 4). Excitation wavelengths were 488 nm for anti-rabbit Alexa Fluor-488 (Invitrogen), 405 nm for anti-guinea pig Alexa Fluor-405 (Jackson Immuno Research, West Grove, PA, USA), 635 nm for anti-mouse Alexa Fluor-647 (Life Technologies, Darmstadt, Germany) and 515 nm for yellow fluorescent protein (YFP; Nagai et al. 2002), respectively. The images were acquired through separate channels and temporally non-overlapping excitation of the fluorochromes and analyzed off-line using ImageJ software package (courtesy of W.S. Rasband, U. S. National Institutes of Health, Bethesda, Maryland, http://rsb.info.nih.gov/ij/). The analysis of co-localization was performed in Fiji/ImageJ software based on the isodata algorithm (Ridler & Calvard 1978) using the auto-threshold plugin.

To compare SNAP47 fluorescence signal intensity and cytoplasmic area in GABAergic INs and glutamatergic neurons between non-infected, scrambled and knockdown groups, we determined the mean labeling intensity over the somata of YFP-VGAT-positive and YFP-VGAT-negative neurons and compared these to the mean labeling intensity of the surrounding astrocytic layer, respectively (as showed in Fig. 2E). All image processing and analysis were performed using Fiji/ImageJ.

Quantification of MAP2-positive processes in GABA- and glutamatergic neurons in three groups (non-infected, scrambled and knockdown) using the ImageJ plugin was used to determine total dendritic length. MAP2-positive dendrites were reconstructed and quantified using a custom ImageJ plugin—SNT (a unifying toolbox for quantification of neuronal anatomy).

Reconstruction and quantification of axonal processes in GABAergic INs was performed in the same manner as for MAP2. However, it was based on YFP-VGAT transgene expression (515 nm for yellow fluorescent protein).

To determine synaptic processes in both types of neurons and for each group (non-infected, scrambled and knockdown), we quantified pre- and postsynaptic protein puncta for GABA (VGAT and gephyrin, respectively) and glutamatergic (VGLUT1 and PSD95, respectively) autaptic neurons.

### Electrophysiology

Whole-cell patch-clamp recordings were performed on hippocampal autaptic neurons between days in vitro (DIV) 14 and 16 at room temperature (RT). Single neurons on micro-islands were selected by its nuclear red fluorescence protein (RFP) signal and recorded using a Multiclamp 700B amplifier (Molecular Devices) and an Axon Digidata 1550 digitizer (Axon Instruments).

The series resistance was compensated by 70% and only cells with series resistances of < 12 MΩ were analyzed. Data were acquired at 10 kHz using pClamp 10 software (Molecular Devices) and filtered using a low-pass Bessel filter at 3 kHz. Data were analyzed offline using Axograph X (Axograph Scientific). Borosilicate glass pipettes with resistance between 2 and 4 mΩ pulled with a micropipette puller device (Sutter Instruments) were used. The patch pipettes were filled with high chloride intracellular solution containing 136 mM KCl, 17.8 mM HEPES, 1 mM EGTA, 0.6 mM MgCl_2_, 4 mM ATP-Mg, 0.3 mM GTP-Na, 12 mM phosphocreatine, and 50 units/mL phosphocreatine kinase (300 mOsm, pH 7.4). The recording chamber was constantly perfused with extracellular solution containing 140 mM NaCl, 2.4 mM KCl, 10 mM HEPES, 2 mM CaCl_2_, 4 mM MgCl_2_, and 10 mM glucose (pH adjusted to 7.3 with NaOH, 300 mOsm).

Extracellular solution containing 3 µM of the AMPA receptor antagonist 2,3-dioxo-6-nitro-1,2,3,4-tetrahydrobenzo[f]quinoxaline-7-sulfonamide (NBQX) was used to distinguish glutamatergic from GABAergic neurons. NBQX and extracellular solutions were applied using an 8-valve (pinch), computer-controlled, fast-flow perfusion system (Automate Scientific Inc). mIPSC and mEPSC were detected for 20 s. For each cell, data were filtered at 1 kHz and analyzed by using template-based mEPSC and mIPSC detection algorithms implemented in AxoGraph X (AxoGraph Scientific). The threshold for detection was set at three times the baseline SD from a template of 0.5 ms rise time and 2 ms decay for mEPSc and 0.5 ms rise time and 18 ms decay for mIPSC. EPSCs and IPSCs were evoked by 2 ms somatic depolarization from − 70 to 0 mV producing an unclamped axonal AP.

All neurons were voltage-clamped at − 70 mV for the recording of miniature events. Under these conditions, both mEPSCs and mIPSCs are inward due to the high intracellular chloride concentration. To reliably discriminate excitatory or inhibitory miniature events, we took advantage of their distinct kinetic properties and pharmacological sensitivity. In particular, glutamatergic mEPSCs were identified based on their fast rise and decay kinetics and their sensitivity to AMPA receptor antagonists (NBQX). While GABAergic miniature events slow rise and long decay kinetics and their insensitive to AMPA receptor antagonists (NBQX). In contrast, GABAergic mIPSC exibited slower rise and decay kinetics and were insensitive to NBQX.

### Statistics

For morphological experiments data were collected from at least 3 independent hippocampal autaptic cultures. All reported values are expressed as normalized standard error of the mean ± SEM. The distribution of the data is presented as violin plots.

To quantify morphological data, we used Dunn's multiple comparison test between different groups and also the Mann–Whitney test for unpaired data. For puncta density, we used the two-tailed Mann–Whitney test for unpaired data. Comparisons between subjects were considered significant if the *P* value was < 0.05 using GraphPad Prism 7.

For quantifying electrophysiological data, we obtained an equal number of recordings in the range of 8 to 10 neurons per experimental group in each replicate culture. Electrophysiological data from 5 independent cultures were normalized to the mean value of the scramble group. Statistical significance was determined by using the two-tailed Mann Whitney test for unpaired data at the indicated significance level (*p < 0.05, **p < 0.01, ***p < 0.001) using GraphPad Prism 7.

## Discussion

In our study, we found that shRNA can efficiently reduce endogenous SNAP47 gene expression in cultured hippocampal neurons. Furthermore, the reduced SNAP47 expression is associated with morphological changes and reduced synaptic connections in GABAergic INs, but not in glutamatergic principal cells, and significantly affects GABAergic transmission in hippocampal neurons.

Our previous study demonstrated strong labeling of SNAP47 protein in VGAT-YFP-positive GABAergic INs compared to the VGAT-YFP-negative glutamatergic hippocampal neurons. However, SNAP47 protein appeared to be trafficked to both pre- and postsynaptic sites in both GABAergic INs and glutamatergic hippocampal principal cells (Münster-Wandowski et al. 2017). The unexpectedly strongest somatic expression of SNAP47 particularly in GABAergic neurons, raised questions regarding its possible functions, that led us to explore the function of this highly expressed and somatically accumulated SNAP isoform in this specific neuron type. Therefore, we developed a lentiviral-based SNAP47 gene knockdown to modulate the expression levels of the protein in the two main populations of hippocampal neurons – inhibitory GABAergic and excitatory glutamatergic and to determine whether this affects their morphology and neurotransmission. Our results demonstrate the impact of SNAP47 knockdown on GABAergic inhibitory neurons, with no apparent effect on excitatory glutamatergic neurons. In fact, SNAP47 knockdown was found to significantly alter the morphology of GABAergic neurons: resulting in reduced soma size and total dendrite length, as well as lower expression of pre- and postsynaptic markers. These morphological changes also have functional consequences, resulting in a decrease in inhibitory synaptic transmission. Although rescue experiments to reintroduce SNAP47 cDNA via a lentiviral system were not performed in this study — in order to provide an additional level of SNAP47 specificity and rule out off-target effects — our conclusions are supported by the specificity of the morphological and functional phenotypes observed specifically in inhibitory neurons following SNAP47 inhibition.

A key question that arises from our current results is why only GABAergic neurons exhibit a significant change in morphological phenotype, and impaired neurotransmission upon SNAP47 silencing?

### Silencing SNAP47 Exclusively Alters the Morphology of Hippocampal GABAergic Interneurons

Based on the substantial somatic accumulation of SNAP47 observed in hippocampal GABAergic neurons in our previous study (Münster-Wandowski et al. 2017), and the documented somatic localization of SNAP47 in cultured striatal neurons (Holt et al. 2006), it is reasonable to conclude that this SNAP47 isoform may have a primary somatic function in inhibitory neurons, potentially involving distinct somatic processes compared to those in glutamatergic neurons.

In general, SNAP47 is expressed much earlier than synaptic proteins during brain development, in a pattern consistent with a "housekeeping" role rather than a specialized synaptic function (Holt et al. 2006). The large somatic pool of SNAP47, as well as trafficking to pre- and postsynaptic compartments in GABAergic INs, suggests an involvement of this protein in local secretory trafficking. Local secretory pathways have been reported for both dendrites and axons of neurons (reviewed in Horton and Ehlers 2004; Kennedy and Hanus 2019). However, to date, Golgi structures have been identified in dendrites, but not in axons, in the central nervous system (CNS). Therefore, the somatic localization and our current finding of reduced somatic size and reduced dendritic length in GABA INs suggest a link of SNAP47 to factors involved in regulating the structural organization of the ER or Golgi-ER and post-Golgi transport, possibly through interaction with cytoskeletal elements. Indeed, Kuster et al. (2015) showed that SNAP47 preferentially interacts with the trans-Golgi network VAMP4 and post-Golgi VAMP7 and −8. Moreover, silencing of SNAP47 further shifted the subcellular localization of VAMP4 from the Golgi apparatus to the ER. The authors concluded that SNAP47 generally plays a role in the proper localization and function of a subset of VAMPs, likely by regulating their trafficking through the early secretory pathway. However, in this study, inhibitory and excitatory neurons were not distinguished.

In fact, the function of SNAP47 originally proposed was a participation in a basic intracellular fusion reaction common to all cells (Holt et al. 2006). One of the most important homeostatic intracellular fusions occurs during somatic autophagy. In response to diverse stimuli, autophagosome formation is initiated, which subsequently undergoes fusion with lysosomes for the degradation of substrates (Zhao et al. 2021). There is increasing evidence that SNAP47, a protein with partial homology to SNAP29, has an important role in autophagic flux (Corona et al. 2018). Consistently, knockdown of SNAP47 inhibits autophagocytic degradation (Corona and Jackson 2018; Corona et al. 2018), possibly by inhibiting fusion of the endosome to autophagosomes, a function that overlaps with that of SNAP-29 (Itakura et al. 2012).

Recent research further suggests that the early secretory pathway, including de ER and Golgi apparatus, plays an important role in the regulation of autophagy beyond providing membrane sources for autophagosome formation. SNAP47 has been shown to colocalize with ER-Golgi intermediate compartment (ERGIC) markers and facilitates the proper localization of VAMP4 and VAMP7 (Kuster et al. 2015). Jian et al. (2024) recently demonstrated an interaction of SNAP47 with post-Golgi VAMP7 and VAMP8, and have documented that the STX17-SNAP47-VAMP7/VAMP8 complex can function as a SNARE complex to mediate the fusion of autophagosomes and lysosomes in mitophagy. Subsequent research revealed that SNAP47 undergoes acetylation and subsequent deacetylation in response to the induction of bulk autophagy or mitophagy (Jian et al. 2025). The requirement of autophagy for proper brain function depends on cell type-specific functions in different neuronal subpopulations. Unfortunately, the physiological role of autophagy has been investigated in glutamatergic neurons, and little is known about these processes in INs. Nevertheless, INs have already been implicated in the pathology of congenital diseases of autophagy, such as tuberous sclerosis complex, epilepsy, developmental delay, and autism (Ridler et al. 2007; Henske et al. 2016). The significant high somatic expression of SNAP47 in GABAergic INs (Münster-Wandowski et al. 2017) and the reduction in the cytoplasmic area of GABAergic INs following SNAP47 knockdown, as observed in the present study, could be due to this protein’s involvement in the autophagy processes. Indeed, it is also known that GABA can increase the number of autophagic vesicles (Kim et al. 2018). Moreover, autophagy has been recently implicated in the regulation of inhibitory neurotransmission in parvalbumin (PV)-expressing neurons (Chalatsi et al. 2022). It remains to be investigated how SNAP47 deficiency leads to the degradation of synaptic trafficking following autophagy in INs and how this is reflected in the morphology, for example the number of ER, mitochondria and other lysosomal structures, since it is known that mitochondria regulate lysosomal dynamics and vice versa (Deus et al. 2020).

The reduction in total dendritic length in GABAergic INs after SNAP47-KD correlates with the reduction in MAP2 expression in our study. MAP2 is exclusively enriched in dendrites and neuronal perikarya and serves to stabilize microtubule (MT) growth by cross-linking MT with intermediate filaments and other MTs. Therefore, reduced MAP2 expression after SNAP47-KD may explain the reduced cytoplasmic area and overall cell body size. This relationship has been discussed before, but rather in the context of neurodegenerative diseases (Mages et al. 2021). In general, MAPs were identified as binding partners for ionotropic GABA_A_ and GABA_C_ receptors decades earlier (Hanley et al. 1999; Wang et al. 1999; Passafaro and Sheng 1999). The inhibitory receptors appear to interact with a different class of intracellular proteins than the excitatory amino acid receptors. Ionotropic GABA and glycine receptors associate with microtubule-binding proteins, whereas NMDA and AMPA receptors associate with PDZ-containing proteins. Binding to specific MAPs may therefore be the mode of cytoskeletal attachment for inhibitory ionotropic receptors in general. A role for microtubule-binding proteins in cytoskeletal anchoring of GABA and glycine receptors may be related to the fact that inhibitory synapses form predominantly on the shafts of proximal dendrites, where microtubules are abundant, rather than on dendritic spines, where microtubules are sparse or absent. Direct alterations of MAP2 structure in endosomal trafficking by SNAP47 silencing could further affect not only the amount of MAP2 expressed, but also the site-specific phosphorylation of MAP2, which is known to affect its microtubule binding, polymerizing, and stabilizing capabilities (Lyu et al. 2025) and could therefore contribute to the regulation of the dendritic microtubule cytoskeleton. MAP2 is necessary along with other intracellular components, e.g., actin, neurofilaments, and mitochondria, to maintain neuroarchitecture. Protein trafficking through the cytoskeleton and motor system, primarily by movement on microtubules, is critical for autophagosome-lysosome encounter and fusion (Jiang et al. 2014). Reduced MAP2 expression induced by SNAP47-KD could negatively affect mitophagosome morphogenesis and autophagosome-lysosome fusion in GABAergic neurons. However, further studies are needed to better understand the impact of MAP2 reduction by SNAP47-KD on somatic processes, dendritic morphogenesis and microtubule function in GABAergic INs.

We observed a close relationship between SNAP47 and MAP2 during neurite outgrowth in inhibitory neurons, since silencing of SNAP47 significantly reduced the expression of MAP2, which is known to be essential for dendritic growth by selectively stabilizing dendritic microtubules (Harada et al. 2002). In the absence of MAP2 in neurons, protein kinase A (PKA) is not sufficient to anchor all PKA molecules released from the MT-rich region (Teng et al. 2001, Harada et al. 2002). However, in the axonal compartments, the amount of PKA did not change significantly as in the dendritic compartments (Harada et al. 2002). This implies that other catalytic subunits appear to be designed to anchor PKA to MAP2 in dendrites and to other anchoring proteins in axons. In fact, the reconstruction and measurements of the axon in GABAergic INs after SNAP47-KD in the present study did not show a significant reduction in axon length. In axons, microtubules are oriented with plus ends out, whereas in dendrites, microtubule orientations are mixed (Yau et al. 2016). The stabilization of microtubules in axons during the establishment of neuronal polarity requires axon-enriched MAP6 (Tortosa et al. 2017). MAP6 is present at Golgi membranes and secretory vesicles in developing neurons, controls axon maturation in vitro and in vivo (Tortosa et al. 2017, Gomis-Rüth et al. 2008, Witte et al. 2008), has strong microtubule stabilizing activity, during neuronal polarization (Cai et al. 2009, Nakata and Hirokawa 2003), and promotes organelle trafficking in axons. The lack of an effect on axon length in GABAergic SNAP47-KD neurons, documented in our study, suggests that bidirectional, anterograde and retrograde axonal transport remains coordinated. Furthermore, palmitoylation of the N-terminal domain controls MAP6 distribution in neurons (Tortosa et al. 2017). During neuronal development, neurite outgrowth as well as axon determination, polarity establishment and axon pathfinding rely on several palmitoylated proteins (Holland and Thomas, 2017). In fact, palmitoylation is known to play a central role in AIS formation and function. However, SNAP47 is an atypical Q-SNARE that has a long N-terminal extension and lacks cysteine palmitoylation-mediated membrane targeting (Kuster et al. 2015). This may also explain the lack of effect on axon outgrowth in cultured GABAergic neurons. Although we did not measure the axon length of the glutamatergic neurons, as their somatic, dendritic and synaptic morphology remained unchanged, a previous study (Shimojo et al. 2015) already indicated, that only around 1% of the total population of SNAP47-deficient neurons in cultures prepared from the cortices of electroporated embryos had slightly longer total axons.

Reduced dendritic length and reduced MAP2 expression due to SNAP47 knockdown was associated with reduced levels of pre- and postsynaptic markers in GABAergic hippocampal neurons. We observed significantly reduced VGAT and gephyrin in GABAergic neurons, while VGLUT1 and PSD95 remained unchanged in glutamatergic neurons. Together with the observed morphological changes in GABA neurons, the reduction in soma size and dendrites, as well as the decrease of MAP2 after KD, the observed reduction in the number of postsynaptic markers such as gephyrin becomes clear. Previous studies have shown that gephyrin, the main organizer of ligand-gated ion channels at inhibitory synapses, interacts with the MT-based cytoskeleton at postsynaptic densities (Kirsch et al. 1991; Kirsch and Betz 1995). Various other studies have demonstrated the important role of gephyrin-MT interactions in postsynaptic cluster formation (Groeneweg et al. 2018), and Zhou et al. (2021) provided additional evidence for gephyrin binding to MTs in brain tissue and in in vitro cell systems.

However, in addition to the reduced amount of gephyrin, we also observed a decrease in the level of the presynaptic marker VGAT in GABAergic INs, despite the length of the axons remaining unchanged. The trafficking of SNAP47 to presynaptic sites has previously been documented in our study and by Shimojo et al. (2015). The latter study discussed the presynaptic role of SNAP47 in the context of BDNF exocytosis from callosal axons. The unchanged axon length and the gephyrin-MT interaction discussed above suggest that the loss of the presynaptic VGAT marker is associated with a gephyrin-dependent loss of synapses. This is supported by an earlier observation by Marchionni et al. (2009), who found that a reduction in gephyrin and in synaptic γ2 subunit-containing GABA_A_ receptor clusters was accompanied by a decrease in VGAT density. Axon-length-independent synapse loss is most severe in inhibitory neurons and is dependent on GABA signaling (Wu et al. 2012). However, the specific mechanisms of SNAP47-mediated synapse loss, particularly those that are axon-length-independent, remain unclear. Our results suggest that a reduced amount of VGAT is a consequence of reduced MAP2 and gephyrin protein puncta and not a local effect of SNAP47 knockdown. SNAP47 does not seem to be directly involved in the outgrowth of axons and in the number of presynaptic components in GABAergic INs. However, the total number of synapses needs to be measured in future to confirm that KD of SNAP47 did not disrupt the formation of GABAergic terminals directly.

Overall, our results showed that SNAP47-dependent alterations occurred exclusively in the morphology of GABAergic inhibitory neurons, with no changes observed in glutamatergic neurons.

### Knockdown of SNAP47 Significantly Reduces Inhibitory Synaptic Transmission but Has No Effect on Glutamatergic Transmission

Our documented SNAP47-dependent morphological alterations in GABAergic neurons, such as a reduction in somatic cytoplasmic area and total dendritic length, as well as a reduction in pre- and postsynaptic markers, strongly suggests impaired inhibitory synaptic transmission.

A change in the number of active synapses should be reflected in spontaneous, miniature IPSC frequency. Indeed, we observed a significantly reduced mIPSC frequency in hippocampal GABAergic INs after SNAP47 silencing. However, no significant change in GABA-mIPSC amplitudes was observed. The reduced mIPSC frequency observed in this study, in the absence of any change in amplitude, suggests that alterations are occurring in either presynaptic function or the number of synapses rather than in the strength of individual synapses at the postsynaptic level. We note, however, that a more detailed analysis of mIPSC kinetics (e.g., rise and decay times) could provide additional resolution in distinguishing pre- versus postsynaptic contributions. Because these parameters were not examined in the present study, this remains an important direction for future work. Accordingly, although our data are consistent with a presynaptic effect, we cannot fully exclude the possibility of subtle postsynaptic contribution. More specifically, variations in mIPSC frequency upon SNAP47 silencing can be associated with reduced GABA vesicle release probability, altered proteins involved in vesicle docking and fusion, or a decrease in the number of functional inhibitory synapses. Although SNAP47 does not participate in all exocytic events, it has been demonstrated that it colocalizes with a subset of VAMP2-mediated exocytic events in developing neurons, thereby altering the fusion mode (Urbina et al. 2021). The E3 ubiquitin ligase TRIM67 controls the exocytic process by decreasing the amount of SNAP47 protein and restricting its incorporation into SNARE complexes. The specific subcellular localization of SNAP47 suggests that it plays a distinct role in regulating the fusion process. It localizes to the endoplasmic reticulum, the Golgi apparatus and post-Golgi compartments (Kuster et al. 2015). This suggests that TRIM67 and SNAP47 may identify sites for the release of secretory vesicles and control modes of exocytosis. However, further investigation is needed to establish whether TRIM67 and SNAP47 are also involved in regulating exocytosis and fusion dynamics in GABAergic synapses, and how they might contribute to the plasticity of these synapses. Electrophysiological measurements of GABAergic synaptic transmission are consistent with our morphological observations, which reveal alterations to GABAergic synapses in vitro and a notably diminished number of pre- and postsynaptic markers. Changes in GABAergic neuron morphology, followed by alterations in the frequency of mIPSCs, can significantly impact neuronal activity and network function. At an in vivo level, these alterations can lead to network dysfunction, affecting processes such as learning and memory, as well as disease development. A decrease in mIPSC frequency reduces inhibitory synaptic transmission and can result in hyperexcitability.

In contrast, reducing SNAP47 levels had no significant effect on the frequency or amplitude of spontaneous miniature EPSCs. This is consistent with published data and suggests that SNAP47 is not essential for maintaining basic excitatory synaptic transmission in glutamatergic neurons. KD of SNAP47 did not affect basal AMPAR- or NMDAR-mediated synaptic responses (Arendt et al. 2015; Jurado et al. 2013).

Based on the observed reduction in mIPSC frequency, we initially hypothesized that evoked IPSCs would also show a decrease in amplitude, reflecting a reduction in the total evoked current. This expectation aligns with the idea that fewer active inhibitory synapses, or a lower probability of vesicle release, would lead to a diminished overall inhibitory response under stimulation conditions. Indeed, our observation of a significantly decreased IPSC amplitude (see Fig. 5D) supports this hypothesis, indicating a reduced efficacy of inhibitory synaptic transmission and a weaker overall inhibitory drive within the network. However, a reduction in IPSC amplitude can also be attributed to possible alterations at postsynaptic level. At the postsynaptic level, reduced IPSC amplitude generally indicates reduced responsiveness, which can result from a lower number of functional GABA receptors. The postsynaptic neuron with fewer GABA receptors, allow less current flow in response to GABA release (Sallard et al. 2021). This implies that SNAP47, as a protein involved in vesicle fusion, might be specifically required for the exocytosis of vesicles carrying GABA_A_ receptors. Alternatively, reduced IPSC amplitude could result from GABA receptors that are less sensitive or exhibit altered properties. This could result in a smaller current, even when the amount of GABA released is normal.

However, our morphological data suggest that the presynaptic mechanisms also contribute. We observed a reduction in VGAT puncta (see Fig. 4B), suggesting that presynaptic neurons may release less GABA. Mechanistically, this decrease likely reflect presynaptic factors, most prominently a lower number of functional inhibitory synapses. Although a reduced number of neurotransmitter-containing vesicles could theoretically contribute, the unchanged amplitude of miniature IPSCs argues against this possibility.

Therefore, our electrophysiological results suggest that the observed effect is most likely due to a lower number of functional inhibitory synapses. This conclusion is also supported by the morphological data. At the network level, the resulting decrease in IPSC amplitudes may compromise inhibitory control, indicating that SNAP47 plays a critical role in modulating GABAergic function and potentially altering neural circuit processing, which could contribute to cognitive or behavioral changes in vivo.

Unlike GABAergic neurons, we observed no changes in glutamatergic neurotransmission. This finding aligns with the published data of Jurado et al. (2013), who demonstrated that SNAP47 KD does not affect the EPSC. The SNAP47 protein does not influence the regulation of basal AMPAR- or NMDAR-mediated synaptic responses or basal AMPAR surface expression. Therefore, SNAP47 appears not to play a role in glutamatergic receptor trafficking.

In summary, the morphological and electrophysiological data presented in this study provide the first indication of synaptic dysfunction in GABAergic SNAP47-KD interneurons. This suggests that this SNAP isoform plays an important role in modulating GABAergic synaptic transmission.

## Conclusion and Future Perspectives

Our study has revealed significant differences in the properties of two main types of hippocampal neurons following SNAP47 knockdown: (1) GABAergic neurons, which depend on SNAP47, and (2) glutamatergic neurons, which appear to be unaffected by the absence of SNAP47 in terms of their normal morphology and function.

The preferential somatic localization of SNAP47 in GABAergic interneurons, alongside the morphological and functional alterations observed following SNAP47 knockdown, suggest that this protein is involved in an early secretory pathway that is specific to interneurons.

However, we were unable to determine whether the phenotype observed in the GABAergic neuron is specifically due to changes in the protein only in the presynaptic site or to changes in the protein only at the postsynaptic site, since the lentiviral infection in our autaptic culture system results in simultaneous manipulation of both compartments. This limitation could be addressed in future studies using complementary approaches, such as sparse infection in mass cultures or paired recordings from single-cell infections, to better unravel the presynaptic and postsynaptic functions of SNAP47.

In line with the limitation described above, because SNAP47 knockdown was induced at early developmental stages (DIV1) and phenotypes were assessed at later time points (DIV14–16), the observed effects may reflect, at least in part, developmental alterations rather than exclusively acute roles in mature synaptic function. Addressing the acute role of SNAP47 in mature synapses would require a different experimental strategy, for example lentiviral delivery of SNAP47 shRNA at later in vitro stages (approximately DIV10–12), when synapses are already fully formed in an autaptic neuronal system.

In the long term, it would be interesting to investigate the impact of SNAP47 KD's selective effect on the morphology and transmission of hippocampal GABAergic neurons in neurodegenerative processes.

## Supplementary Information

Below is the link to the electronic supplementary material.

ESM 1 (PDF 583 KB)

ESM 2 (TIF 8.00 MB)

ESM 3 (TIF 8.00 MB)

ESM 4 (TIF 8.00 MB)

## Data Availability

All data generated or analyzed during this study are included in this article (and its supplementary information files). Additional data are available from the corresponding authors upon reasonable request.
