# Supplementary material for: SNAP47 Silencing Impairs the Morphology and Neurotransmission of Hippocampal GABAergic Neurons
Source: Mol Neurobiol. 2026 May 15;63(1):632. doi: 10.1007/s12035-026-05907-8 (PMC13179171; doi:10.1007/s12035-026-05907-8)
Supplement: Supplementary file 1 — (PDF 583 KB) [file 12035_2026_5907_MOESM1_ESM.pdf]

Supplementary Information for

Cellular and Molecular Life Sciences

SNAP47 silencing impairs morphology and neurotransmission of hippocampal GABAergic neurons

Zhengzheng He[1] † *, Marcial Camacho[2,3] † *, Thorsten Trimbuch[2,4], Heike Heilmann[1], Christian Rosenmund[2,4], Imre Vida[1] and Agnieszka Münster-Wandowski[1]*

[1]Institute for Integrative Neuroanatomy, Charité - Universitätsmedizin Berlin, Corporate Member of Freie Universität Berlin, Humboldt-Universität zu Berlin, and Berlin Institute of Health, Berlin, Germany
[2]Institute of Neurophysiology, Charité - Universitätsmedizin Berlin, Corporate Member of Freie Uni-versität Berlin, Humboldt-Universität zu Berlin, and Berlin Institute of Health, Berlin, Germany
[3]Department of Physical Medicine and Pharmacology, University of La Laguna: San Cristóbal de La Laguna, Spain
[4]NeuroCure Cluster of Excellence, Charité - Universitätsmedizin Berlin, Campus Mitte, Berlin, Germany

† Zhengzheng He and Marcial Camacho contributed equally to this work

*Correspondence:

zhengzheng.he@charite.de

mcamachp@ull.edu.es

agnieszka.muenster-wandowski@charite.de

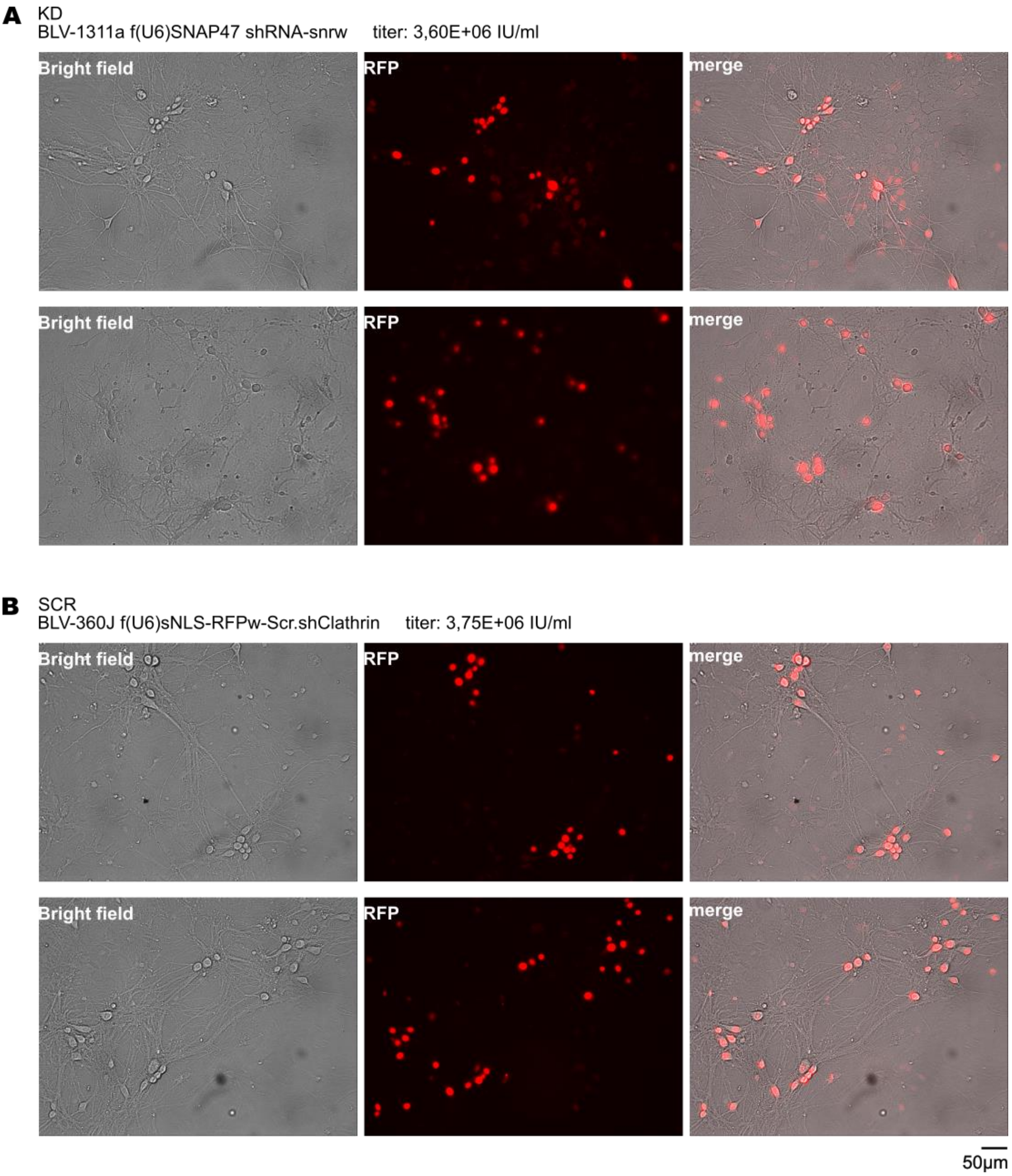

Supplementary Figure S1 Images of the RFP florescent confirming lentivirus in the hippocampal cultures.

A, B: The hippocampal neuronal cultures were treated with 50μl 3,60 X 106 IU/ml of SNAP47-shRNA (KD) and 50μl 3,75 X 106 IU/ml of SCR-shRNA (SCR) lentiviruses, respectively. Two representative images of group KD and SCR from different cultures showed that the neurons were healthy and the nuclear RFP expression was observed in all cultures

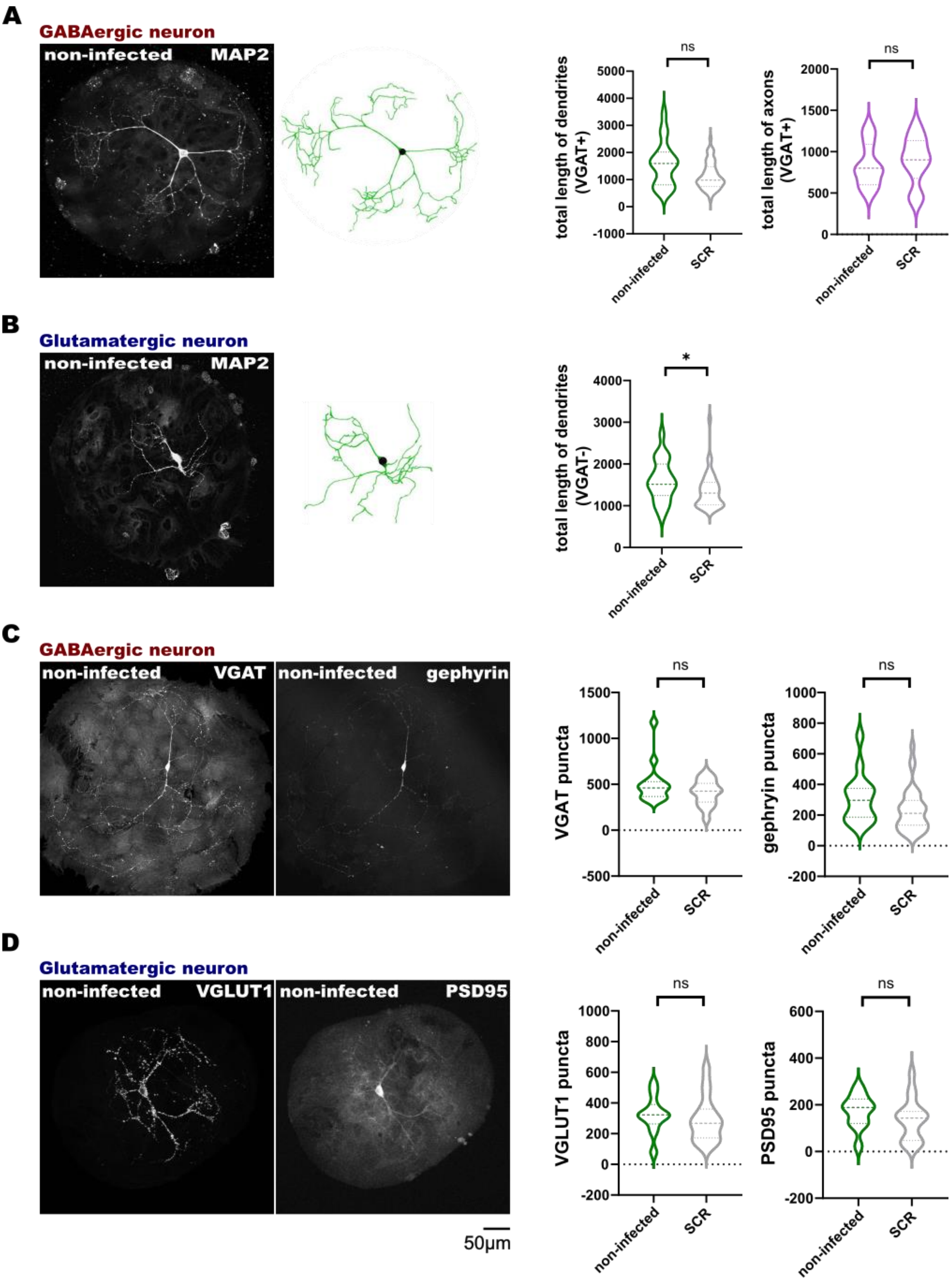

Supplementary Figure S2 Representative morphology and quantitative data in two neuron types and their pre- and post-synaptic markers.

A: The MPA2 labelling and the dendrites reconstruction of the GABAergic neuron in non-infected group. And the quantification of dendrites and axons between non-infected and SCR. (n(non-infected)=19, n(SCR)=50)

B: The MPA2 labelling and the dendrites reconstruction of the glutamatergic neuron in non-infected group. And the quantification between non-infected and SCR. (n(non-infected)=20, n(SCR)=37, p=0.0446)

C, D: Double immunolabeling of pre- and postsynaptic markers for GABAergic and glutamatergic neurons respectively in non-infected group. Note that there is no significant difference between non-infected and SCR in two neuron types. (C: n(non-infected)=15, n(SCR)=35; D: n(non-infected)=11, n(SCR)=16.)

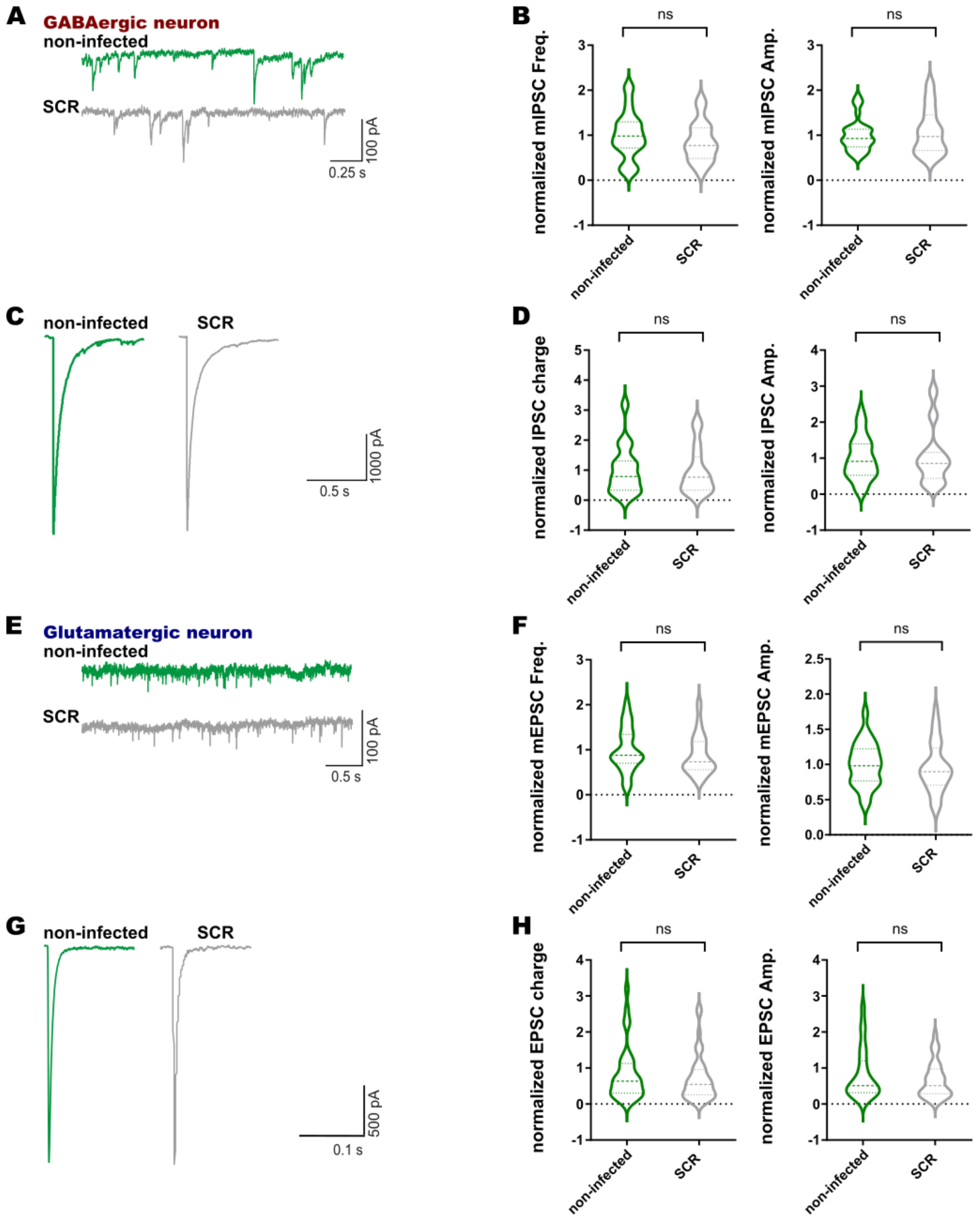

Supplementary Figure S3 Electrophysiological characterization of GABAergic- and glutamatergic synaptic transmission in the group non-infected and SCR.

A, B: Representative patch-clamp recordings of mIPSC and the quantification of the normalized frequency and amplitude in non-infected and SCR. (n(non-infected)=28, n(SCR)=25)

C, D: Representative recordings of evoked IPSC and their measurements of normalized charge and amplitude in non-infected and SCR. (n(non-infected)=28, n(SCR)=23)

E, F: Representative traces of mEPSC and the quantification of the normalized frequency and amplitude in non-infected and SCR. (n(non-infected)=25; n(SCR)=25)

G, H: Representative traces and quantifications of evoked EPSC showing normalized charge and amplitude in non-infected and SCR. (n(non-infected)=25; n(SCR)=30)
