# Supplementary figures and images for: SNAP47 Silencing Impairs the Morphology and Neurotransmission of Hippocampal GABAergic Neurons
Source: Mol Neurobiol. 2026 May 15;63(1):632. doi: 10.1007/s12035-026-05907-8 (PMC13179171; doi:10.1007/s12035-026-05907-8)

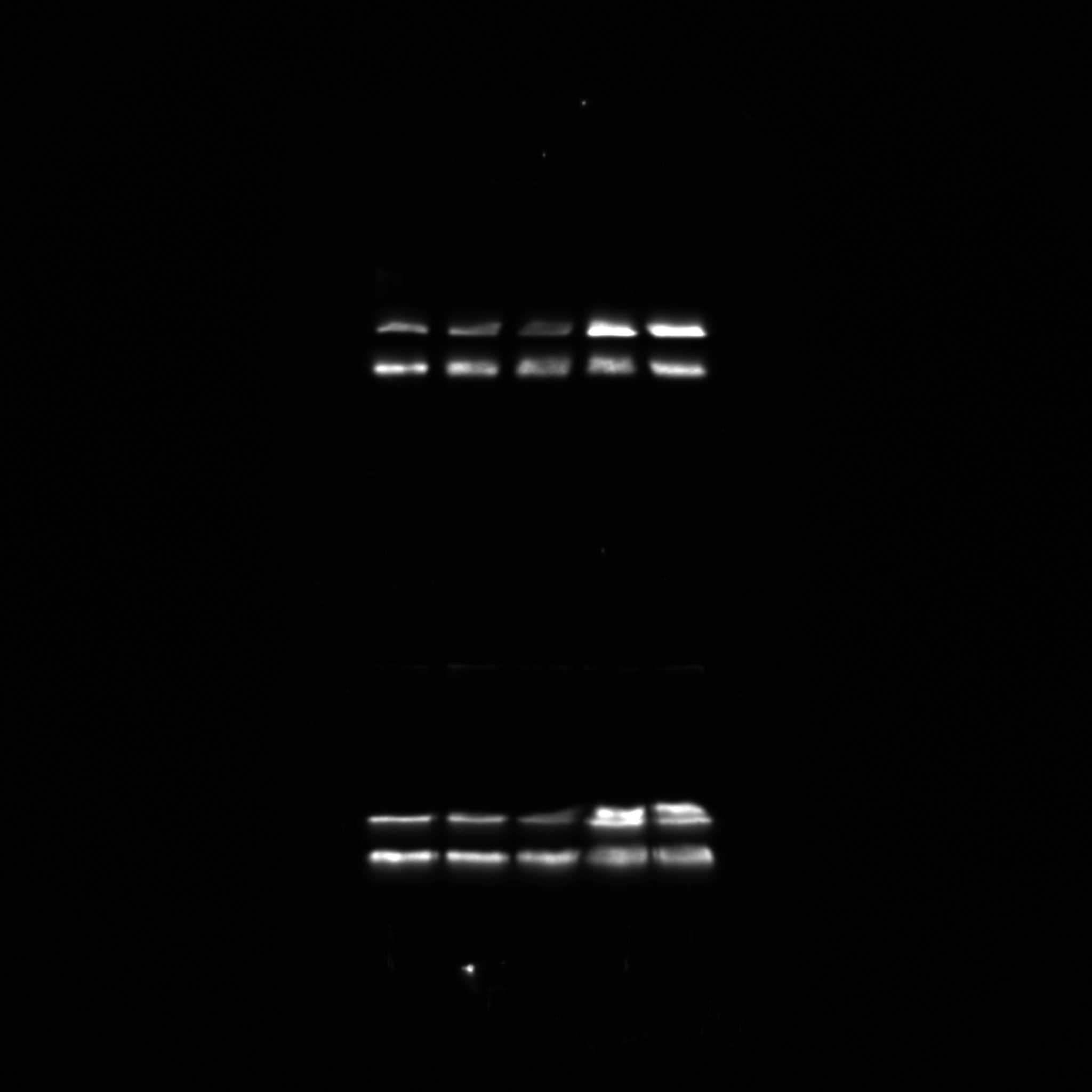

Supplement: Supplementary file 2 — (TIF 8.00 MB) [file 12035_2026_5907_MOESM2_ESM.tif]

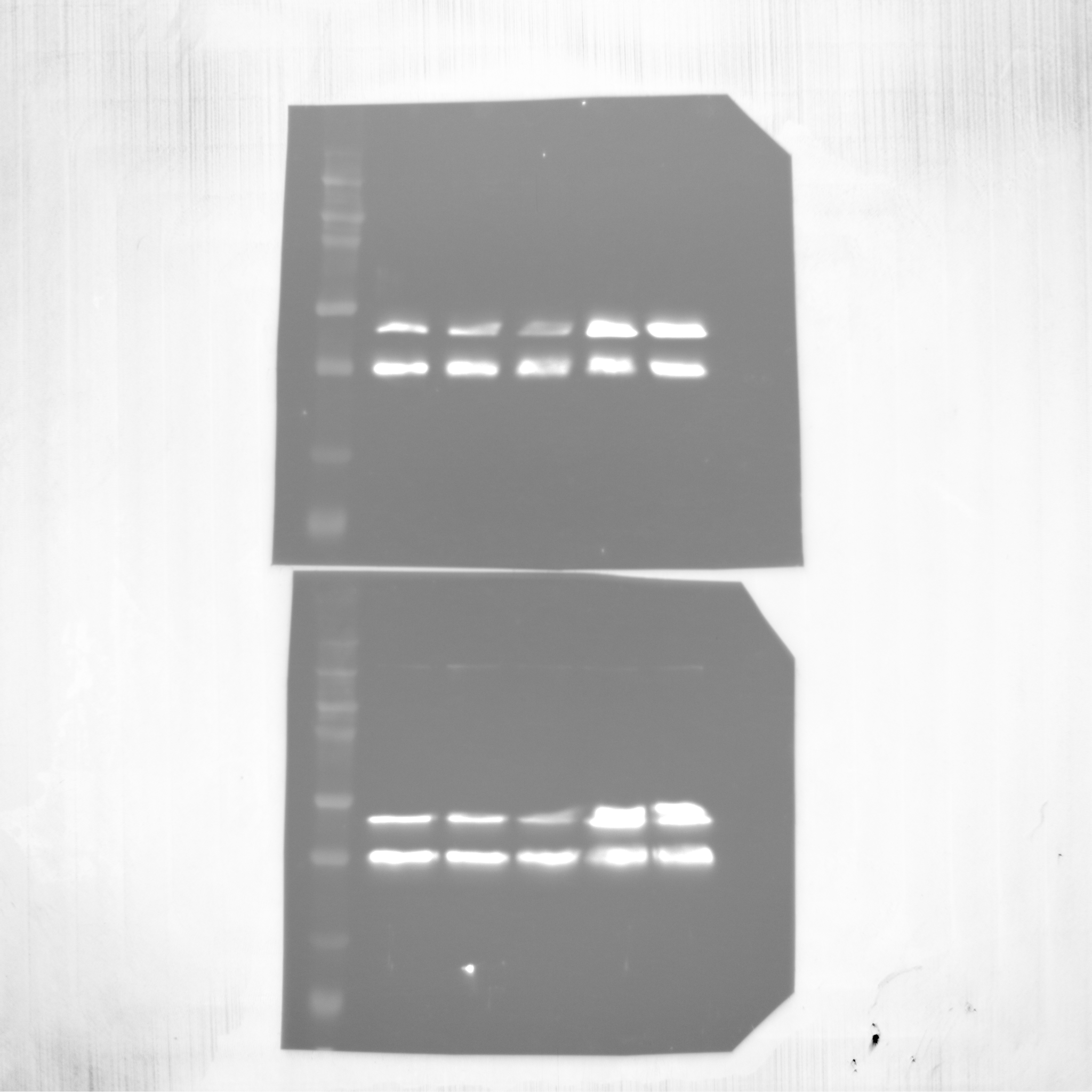

Supplement: Supplementary file 3 — (TIF 8.00 MB) [file 12035_2026_5907_MOESM3_ESM.tif]

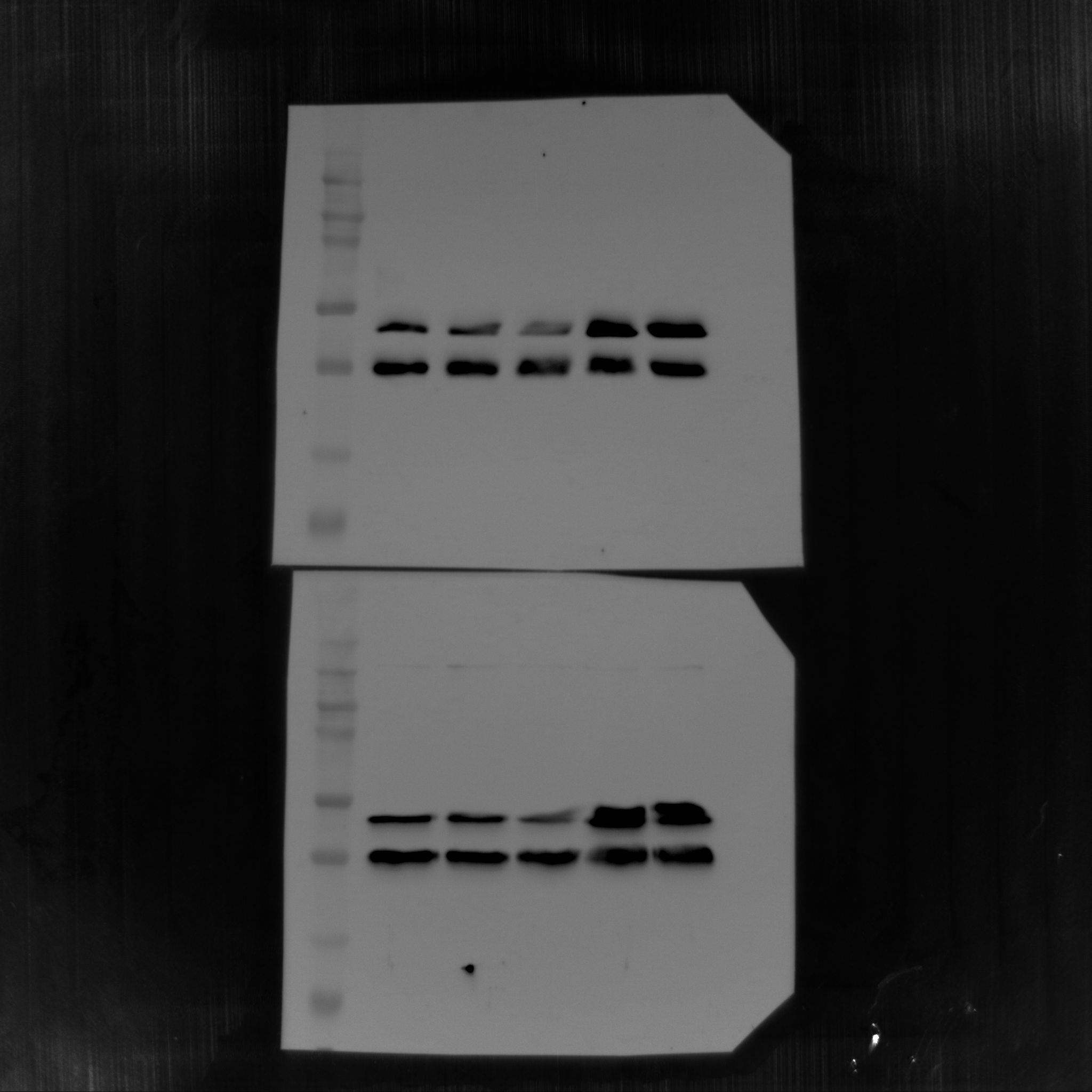

Supplement: Supplementary file 4 — (TIF 8.00 MB) [file 12035_2026_5907_MOESM4_ESM.tif]
